# WASP: a pipeline for functional annotation prediction based on AlphaFold structural models

**DOI:** 10.1038/s41467-026-75956-z

**Published:** 2026-07-28

**Authors:** Giorgia Del Missier, Kiyan Shabestary, Rodrigo Ledesma-Amaro

**Affiliations:** 1 Department of Bioengineering, Imperial College London, London, UK; 2 Bezos Centre for Sustainable Protein, Imperial College London, London, UK; 3 UKRI Microbial Food Hub, Imperial College London, London, UK; 4 Imperial College Centre for Engineering Biology, Imperial College London, London, UK

**Keywords:** Industrial microbiology, Protein function predictions, Metabolic engineering

## Abstract

Protein function annotation is crucial for understanding biological processes and mechanisms. Traditionally, annotations rely on sequence homology, providing valuable insights but often leaving gaps even in well-characterised organisms. With AlphaFold enabling rapid generation of protein structural models, we can now infer function from three-dimensional shape. Here, we present WASP, a pipeline leveraging structural homology to enhance protein annotation prediction at scale, providing a more comprehensive understanding of protein functions across various organisms. WASP relies on network topology for better accuracy and more robust statistical power. We show that WASP achieves superior F1 scores compared to state-of-the-art sequence-based tools when recovering hidden annotations. On 20 industrially relevant organisms, WASP retrieves annotations for 20-30% of previously uncharacterised proteins. We further demonstrate utility in genome-scale metabolic model curation, identifying native candidates for 75-100% of orphan reactions. WASP highlights how structural homology can systematically discover annotations missed by sequence-based approaches.

## Abstract (web summary)

WASP predicts protein functions from AlphaFold structures using network-based structural homology, retrieving annotations for 20-30% of uncharacterised proteins and filling metabolic model gaps by mapping 75-100% of orphan reactions.

## Introduction

Proteins are complex and dynamic macromolecules that are integral to all cellular processes, from metabolic catalysts to providing structural support essential to the cell. The function of a protein is tightly linked to its three-dimensional (3D) shape and protein structural models are critical predictors of protein molecular functions, the effect of mutations, as well as the interactions with other molecules or ligands^1^. The use of artificial intelligence (AI) has marked a paradigm shift in developing protein structural models: deep neural networks such as AlphaFold2^2^, ESMFold^3^ and RoseTTAFold^4^ enable accurate prediction of 3D protein structures with atomic-level precision directly from sequence, massively accelerating protein structural model generation compared to previous time-consuming experimental techniques such as cryo-electron microscopy or X-ray crystallography.

Despite these advancements in structural prediction, genome annotation (or functional annotation) is mainly driven by either sequence homology^5–9^ or through large-scale loss-of-function studies involving knock-out libraries^10,11^, potentially missing on many annotations that could be inferred from structural insight. Even for well-characterised model organisms like *Saccharomyces cerevisiae* and *Escherichia coli*, more than 30% of their gene products are missing a molecular function annotation^12,13^. This annotation gap is also evident in the UniProt Knowledgebase, the primary repository for protein function: while, in its latest release, the full database contains around 250 million sequences, the human-curated subset SwissProt only accounts for 0.2% of these, of which only 9% have experimental validation^14^. Over the past decade, the Critical Assessment of Functional Annotation (CAFA) community challenge has reported notable advancements in protein function prediction algorithms^15^. The most common approaches rely on sequence similarity^16,17^ and homology detection^18,19^. Functional annotations are transferred to newly identified proteins using predefined descriptors, facilitating categorisation into protein families such as Pfam^20^, PANTHER^21^, and CATH^22^. Additionally, ontologies like Gene Ontology (GO) terms^23^, Rhea IDs^24^, and Enzyme Commission (EC) numbers^25^ are often used to provide structured frameworks for functional classification. Despite their successes, for many proteins, annotation through sequence homology remains challenging, with 40% sequence identity often considered as threshold for function conservation, leading to the overlook of more distant evolutionary homologies^26,27^ and those resulting from convergent evolution^28^. Additionally, sequence similarity does not always imply functional equivalence, as seen with proteins resulting from gene duplication but with divergent functions^29^.

**Fig. 1:**
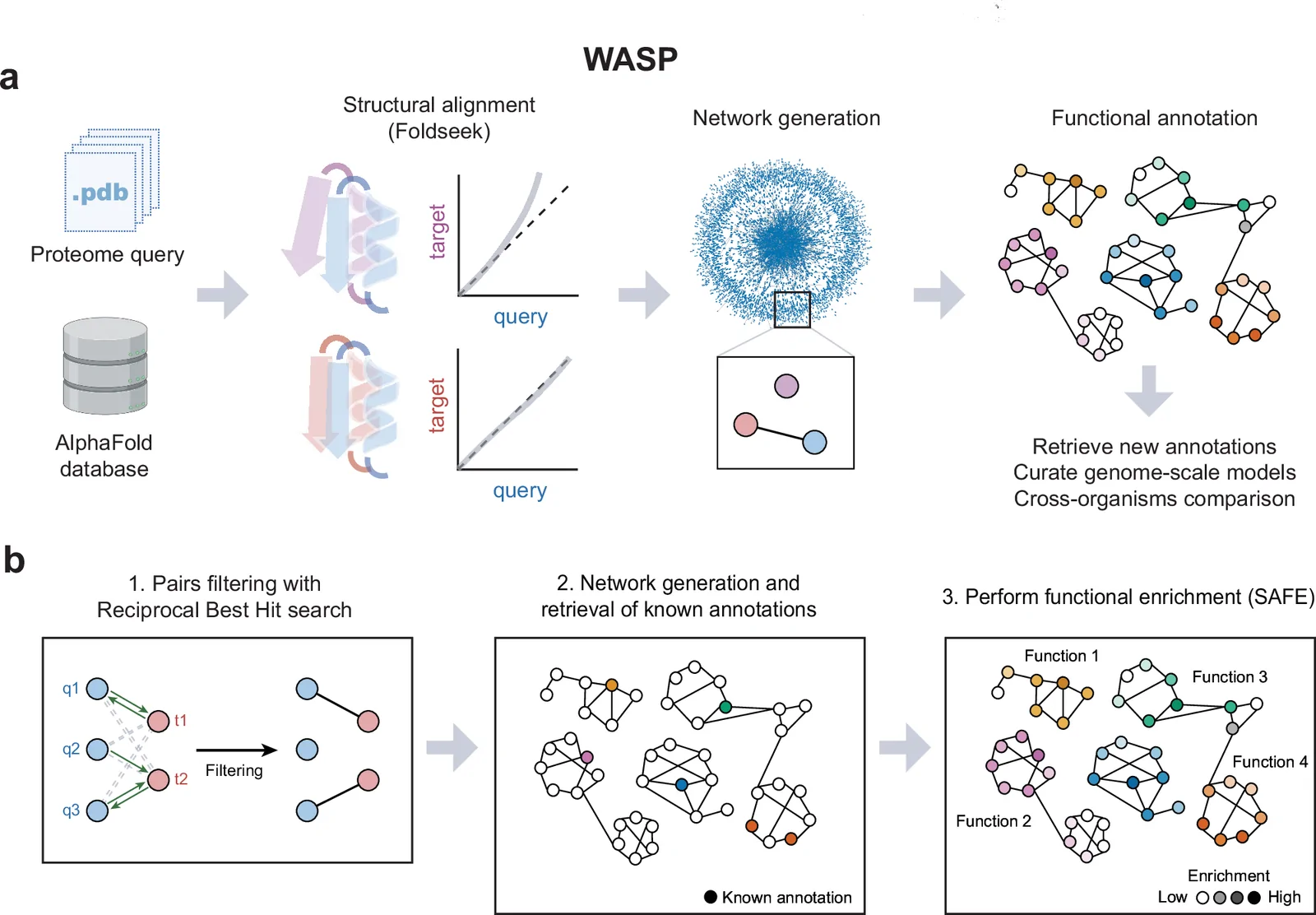
Overview of WASP pipeline. **a** General pipeline where a proteome query is compared to AlphaFold database using Foldseek^30^ structural alignment tool. A network linking the proteome query to AlphaFold database is generated and pruned. Based on network topology, annotations can be made based on structure, which enable the prediction of new functions. **b** Network generation and functional enrichment in WASP. A Reciprocal Best Hit strategy is used to only retain Foldseek hits that are reciprocal between query and target proteins, represented as green bi-directional arrows. When available, functional annotations, such as GO terms, Pfam domains, PANTHER families, CATH superfamilies, Rhea IDs and EC numbers are retrieved and functional enrichment is inferred using network topology principles through SAFE^37^.

Here we present Whole-proteome Annotation via Structural-homology Prediction (WASP), a python-based pipeline designed for comprehensive organism functional annotation prediction (from here on, referred to as annotation) at the whole-proteome level based on structural homology (Fig. 1a). WASP is a user-friendly command-line tool that only requires the NCBI taxonomy ID of the organism of interest as an input. Using the computational speed of Foldseek^30^, WASP generates a graphical representation of reciprocal hits between the organism protein query and the AlphaFold database^31^, enabling downstream robust functional enrichment and statistical testing (Fig. 1b). WASP could annotate previously uncharacterised proteins through multiple functional descriptors such as Gene Ontology (GO) terms, Pfam domains, PANTHER family classification and CATH superfamilies, as well as annotation for specific biochemical reactions, including Rhea IDs and Enzyme Commission (EC) numbers. Additionally, WASP could map native proteins to orphan reactions of genome-scale models, where gene associations had been previously missing, providing insights for fundamental and applied research.

## Results

### A network representation to annotate proteins based on structural homology

**Fig. 2:**
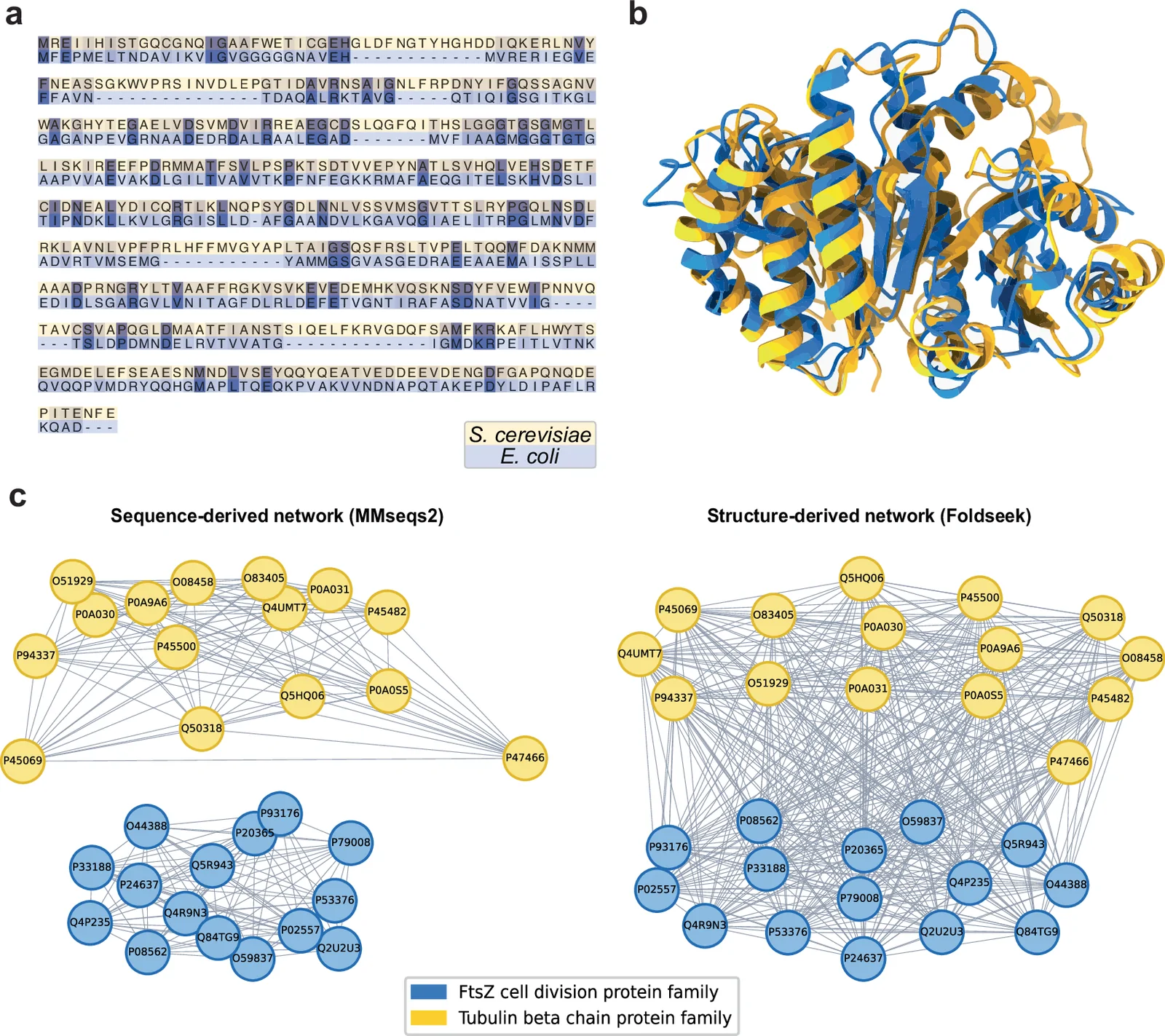
Structural versus sequence-based homology in the FtsZ/tubulin protein family. **a** Sequence alignment between the tubulin beta-chain from *S. cerevisiae* (P02557) and the FtsZ cell division protein from E. coli (P0A9A6). Pairwise darker colours indicate higher amino acid sequence conservation. **b** Structural alignment of the same proteins obtained through Foldseek. The alignment was visualised through ChimeraX^63^. **c** Comparison of the homology networks for a sample of proteins belonging to the FtsZ/tubulin protein family. The sequence-derived network (left) was generated using alignment data from MMseqs2, while the structure-derived network (right) was created using Foldseek. The networks highlight the extensive structural conservation across the family despite sequence divergence.

Protein structure is the major determinant of protein function so that proteins undergoing substantial sequence divergence but a preserved structure exhibit similar functional roles. One example is the small GTPase FtsZ/tubulin protein family, involved in cellular division and maintaining structural identity across all kingdoms of life^32^. Despite the low amino acid sequence homology observed between two representative proteins from *E. coli* and *S. cerevisiae* (around 15% sequence identity, Fig. 2a), and a slightly higher 40% when considering the average between all possible pairs of proteins in SwissProt belonging to the family (Supplementary Data 1 and Supplementary Fig. 1), members of the FtsZ/tubulin family exhibit a very conserved structure. For context, the TM-score measures similarity between structures and is indicated by a number from 0 to 1, with 1 denoting a perfect match^33^. A value of 0.5 is generally considered the threshold for orthology^34^. In the case of the FtsZ/tubulin family, the structural alignment between the two representatives from *E. coli* and *S. cerevisiae* achieves a TM-score of 0.60 (Fig. 2b), which increases to 0.77 when considering the entire family (Supplementary Data 1 and Supplementary Fig. 1). As proof of concept, we used the sequence and structure alignment results for a sample of 15 proteins from the FtsZ/tubulin family to build a graph showcasing similarity relationships (edges) between proteins (nodes). Sequence alignment results produced two separate clusters, while structure alignment resulted in a fully interconnected graph (Fig. 2c). Using this principle of structural conservatism on function, WASP uses structural homology to explore relationships that could not be previously found using sequence homology (Fig. 1b).

First, WASP fetches protein structure models of the query organism from AlphaFold DB (AFDB) hosted on the Google Cloud Bucket. A pre-clustered, indexed version of AFDB and the curated SwissProt database, obtained through the structural alignment tool Foldseek^30^ are also downloaded. Subsequently, Foldseek performs a whole-proteome structural BLAST to identify pairs of homologues between the query organism’s protein models and structures from the two databases. In brief, Foldseek flattens each structure model into a sequence of spatial coordinates (3Di alphabet) that expresses tertiary residue-residue interactions between neighbouring regions. Using this lower level representation, Foldseek reduces the structure comparison task to a simpler sequence alignment: it first employs a fast and sensitive pre-filter algorithm to narrow down potential matches, and then uses a more detailed alignment to achieve the final, optimal superimposition between query and target structures. To further increase confidence in the identified pairs, a Reciprocal Best Hits (RBH) approach is employed to filter out hits that are not bidirectional, that is pairs where the initial query is not among the best hits of the target protein (Fig. 1b; step 1). Commonly used in comparative genomics^35^, RBH was adapted for structural homology and has been shown to allow discovery of more distant homologous protein pairs^36^. WASP also allows for the exclusion of hits above a user-defined sequence identity threshold, facilitating the discovery of functional annotations specifically in the twilight zone of protein evolution where sequence homology is limited.

Second, the retained pairs are used to build a network, where each node represents a protein from either the query organism or the database (target). Edges are weighted according to the alignment scores between the corresponding nodes. This network representation allows us to infer protein function by leveraging not only structure similarities identified by Foldseek, but also the global topology of the constructed graph. Modules and patterns of connectivity between each query, its immediate neighbours and its n-neighbours help in the detection of clusters of homologous structures, which are not as readily deduced when using pairwise alignments alone. Once the network is constructed, the function of uncharacterised proteins can be inferred based on the known function of proteins lying in their n-neighbourhood. WASP retrieves prior existing annotations through the UniProt API^14^ six functional categories: GO terms, Pfam domains, PANTHER families, CATH superfamilies, Rhea IDs and EC numbers (Fig. 1b; step 2).

Finally, the obtained annotation is used by WASP to perform functional enrichment using the SAFE algorithm^37,38^. This method was designed to annotate biological networks using spatial context and was previously used to annotate functionally similar genes from a genetic interaction network^10^. In this step, annotation information is propagated among proteins belonging to the same homology cluster and a hypergeometric test is used to compute a neighbourhood enrichment score. This score represents the likelihood that an uncharacterised protein within the cluster is associated with the same functional descriptor as other proteins in its neighbourhood. In this way WASP measures the protein functional distribution across local neighbourhoods of the network, creating a functional map of the organism’s proteome (Fig. 1b; step 3).

### WASP retrieves hidden labels at high accuracy

We evaluated WASP performance using various datasets and compared it against several state-of-the-art tools for protein functional automated annotation, including EggNOG^6^, CLEAN^7^, DeepEC^8^ and ProteInfer^9^. These tools use different sequence-based techniques and multiple types of functional descriptors to achieve annotation (Table 1).

**Table 1:** Overview of protein functional annotation tools used in WASP benchmark

	Method type	Annotation scope	Functional descriptors
WASP	Structure-based homology groups	Whole-genome	Pfam, PANTHER, CATH, Rhea ID, EC number, GO terms
EggNOG	Sequence-based orthology groups	Whole-genome	Pfam, EC number, GO terms, KEGG pathways
CLEAN	Sequence-based contrastive learning	Individual proteins	EC number
DeepEC	Sequence-based convolutional neural network	Individual proteins	EC number
ProteInfer	Sequence-based convolutional neural network	Individual proteins	EC number, GO terms

Comparison of state-of-the-art tools evaluated in this study to assess WASP performance. Unlike the others, WASP derives annotation starting from structural information and provides a broad range of functional descriptors for a more comprehensive understanding of protein molecular activity.

**Fig. 3:**
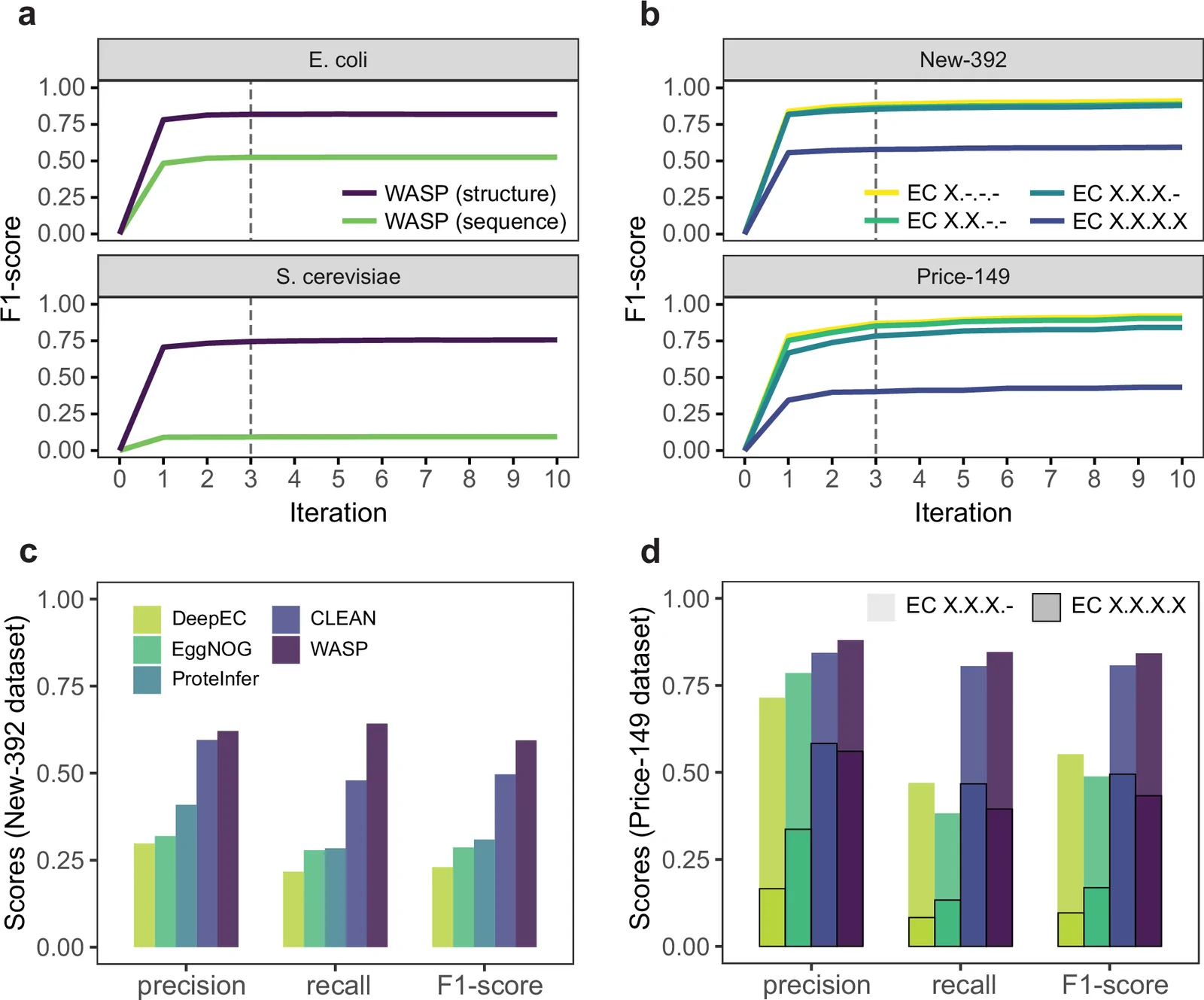
Benchmarking WASP against similar functional annotation tools. **a** F1-score improvement over WASP iterations, averaged over all descriptors for *E. coli* (top) and *S. cerevisiae* (bottom) proteomes. **b** F1-score improvement for the different levels of EC number descriptor for the New-392 (top) and Price-149 (bottom) datasets. **c**, **d** Benchmarking WASP against other annotation tools based on the (**c**) New-392 and (**d**) Price-149 datasets. For benchmarking, 10 iterations of WASP were performed. Scores for ProteInfer in (**c**) were obtained from ref. ^7^. Two test datasets, New-392 and Price-149 from ref. ^7^, were used to benchmark WASP against other annotation tools. In (**d**), filled bars with a bold outline indicate scores for full four-digit EC numbers (X.X.X.X), while filled bars with no outline indicate scores for EC annotations up to the third digit (X.X.X.–). Source data are provided as a Source Data file.

To demonstrate the efficacy of a structural approach in protein automated annotation, we first employ a set of well-characterised proteins for which all functional descriptors are present in UniProt, resulting in the selection of 90% proteins with a UniProt score above 3/5. We selected all proteins meeting these criteria and with an available AlphaFold model in two well-characterised organisms, *Escherichia coli* and *Saccharomyces cerevisiae*, hiding correct labels during WASP execution. During this step, we also explored the impact of multiple iterations of WASP, gradually increasing the number of neighbours included in the network for each query protein from 10 to 100 (ten iterations in total; increasing the number of neighbours by ten at each iteration). Iteratively increasing the number of neighbours helps recover annotations for proteins that are initially unlinked or part of low-connectivity regions by introducing potentially informative associations. This strategy was introduced to find a balance between precision and recall, ensuring that confident annotations are made early, while enabling broader coverage in later steps for proteins still missing a functional prediction. We computed precision and recall scores separately for each of the six functional descriptors in both datasets (Supplementary Figs. 2 and 3), while the harmonic mean of precision and recall, the F1-score, was computed and averaged over all descriptors. WASP consistently achieved high F1-scores (above 80%), showcasing its proficiency in correctly retrieving protein labels (Fig. 3a). Moreover, despite the incremental increase in the number of neighbours over the 10 iterations performed, the rise in false positives was minimal (on average, around 1-2% increase per iteration). A plateau in performance was typically observed within the first two to three iterations, which motivated us to use three iterations as default parameter. Notably, we retained the iterative approach despite the low false positive increase, as it maintains high specificity by prioritising confident, local enrichments before relaxing stringency. All parameters, including the number of iterations and neighbours per iteration, remain customisable by the user to adapt to different annotation goals or dataset characteristics.

WASP performance was also evaluated against a sequence-based version of the pipeline, where network construction and functional enrichment were based on sequence alignments generated by MMseqs2^39^ instead of Foldseek. In this case, F1 scores were notably lower, especially for the *S. cerevisiae* dataset (Fig. 3a). The reduced performance stemmed primarily from the algorithm’s failure to identify reciprocal best hits for inclusion in the network. This resulted in approximately 43% and 90% of proteins being excluded from the analysis for *E. coli* and *S. cerevisiae*, respectively. Notably, the distribution of sequence identity scores compared to structure identity between query organism proteins and their highest-scoring structural hits exhibited substantial variability, especially in *S. cerevisiae* (Supplementary Fig. 4). This likely explains why the same hits were not considered by the sequence-based version of the algorithm and further underscores the challenges posed by sequence divergence when annotating evolutionary distant proteins using amino acid information alone.

We next benchmarked WASP against similar tools for protein function prediction and further evaluated WASP’s ability to classify proteins using EC numbers, the gold-standard functional descriptor, most widely recognised numerical classification system for enzymes and predicted by all the tools considered for benchmarking (Table 1). EC numbers consist of four digits and provide a hierarchical classification scheme, allowing enzymes to be categorised into increasingly specific subcategories. For all tools, we computed or retrieved scores from two independent datasets, referred to as New-392 and Price-149. New-392 comprises 392 enzymes covering 177 EC numbers, with data sourced from SwissProt released after CLEAN was trained (April 2022) and Price-149 is a dataset of experimentally validated results^11^. We computed scores for WASP, EggNOG-mapper, CLEAN and DeepEC. EggNOG-mapper^6^ a sequence-based tool for fast automated annotation of newly sequenced genomes, using precomputed orthologous groups from the eggNOG database to transfer functional information to proteins, while CLEAN is the current state-of-the-art, publicly available tool for EC number prediction based on contrastive learning^7^. DeepEC uses 3 convolutional neural networks, and an additional homology analysis module, for prediction of proteins’ EC number^8^. Scores for ProteInfer performance on the New-392 dataset were retrieved from the original CLEAN study. Moreover, to test whether our topology-driven enrichment adds significant value, we compared WASP against a simpler structural-homology baseline in which each query protein inherits all functional labels from its highest-scoring hit—a strategy commonly used in previous structure-based annotation studies^40–42^.

For both datasets, WASP achieved an average F1 score of over 85% for predicting the 3^rd^ digit of the EC number. Moreover, it attained F1 scores of 60% and 40% for predicting the EC number up to the fourth digit in the New-392 and Price-149 datasets, respectively (Fig. 3b). While the best-hit transfer (referred here as BH transfer) outperforms all methods on the well-annotated New-392 dataset, it collapses on the Price-149 set, highlighting that direct label transfer suffices only when annotations are dense and consistent. In contrast, WASP approach delivered robust performance across both contexts, maintaining high F1-scores even when annotation quality is variable. In fact, WASP outperformed all the other automated annotation tools in precision, recall and F1 score for the task of annotating the New-392 dataset (Fig. 3c). In the Price-149 dataset, WASP achieved similar scores to CLEAN when considering annotation up to the fourth digit (Fig. 3d). However, it outperformed CLEAN across all metrics when considering classification up to the third digit (Fig. 3d). Price-149 dataset was deemed challenging due to the existing sequences being incorrectly or inconsistently labelled by automated methods. Despite relying on annotations from UniProt for the functional enrichment step, these findings demonstrate that WASP is less affected by incorrect annotation thanks to the use of more robust structural information. The difference in methodology between CLEAN and WASP makes the two tools complementary, leading to the correct classification of around 22% and 13% more proteins in the two datasets thanks to the inclusion of structure-based annotation (Supplementary Fig. 5) and leading to a 25% and 30% increase in F1-score respectively when the results from the two tools were combined (Supplementary Fig. 6).

### Improving annotations of industrially relevant organisms

We applied WASP to a cohort of 20 model organisms relevant to industrial applications (Supplementary Data 2). WASP could increase predicted annotations for each functional descriptor across all organisms and particularly excelled in enhancing the annotation of less conventional species, such as *K. pastoris*, *G. metallireducens*, *P. aeruginosa* or *V. natriegens* (Table 2 and Supplementary Data 3). For these organisms, WASP successfully predicted annotations for up to 20-30% of previously uncharacterised proteins. Additionally, WASP also identified annotations in well-characterised model organisms, including *S. cerevisiae* and *E. coli*. In *E. coli*, these proteins typically had low UniProt annotation scores (average score <3), whereas in *S. cerevisiae* most proteins had scores above 4. Despite this, many lacked molecular activity information as described by EC numbers and structural domains provided through CATH, which were effectively recovered by WASP. Examples include *S. cerevisiae* proteins P13045 and P38616. P13045, with a Uniprot score of 4/5 and annotated as a galactokinase, had its predicted annotation augmented by WASP with the corresponding EC number and Rhea ID. Similarly, P38616, despite a score of 4/5, lacked structural domain information and molecular function, which WASP identified as an asparaginase. Additionally, P09932, an uncharacterised protein with a score of 3/5, was predicted by WASP to be a proton/ATP synthase, an annotation supported by the EC diversity reported in CATH for proteins with the same structural domain. In *E. coli*, examples include P77297 and P76148, two uncharacterised proteins with no annotation (score 1/5), that were predicted respectively as a succinate-CoA ligase and a glutaminase (Supplementary Table 1). Overall, WASP demonstrates the ability to standardise annotation levels across all descriptors for both model and non-conventional organisms.

**Table 2:** Predicted annotation obtained through WASP in 20 industrially relevant organisms

	Pfam (%)	PANTHER (%)	CATH (%)	EC number (%)	Rhea ID (%)	GO terms (%)
*S. cerevisiae S288c*	11	9	14	6	6	4
*S. elongatus* *PCC 7942*	17	13	11	17	7	27
*K. pastoris*	21	15	19	18	13	33
*C. reinhardtii*	8	5	8	7	5	8
*S. oneidensis* *MR-1*	18	12	12	11	6	17
*L. lactis subsp. lactis IL1403*	19	16	14	20	7	33
*P. putida* *KT2440*	21	14	16	16	8	29
*C. necator* *H16*	21	16	15	14	10	27
*Y. lipolytica CLIB122*	15	7	15	15	11	13
*C. glutamicum ATCC 13032*	17	16	11	17	7	25
*R. toruloides* *NP11*	28	27	22	15	11	27
*E. coli* *K12*	14	16	18	12	6	16
*G. metallireducens GS-15*	28	19	19	22	8	28
*E. gossypii* *ATCC 10895*	24	14	19	16	13	15
*M. tuberculosis H37Rv*	27	17	18	20	7	31
*V. natriegens*	25	15	14	23	10	29
*P. aeruginosa UCBPP-PA14*	22	16	16	23	9	32
*Synechocystis sp. PCC 6803*	24	14	14	15	5	29
*C. albicans* *SC5314*	24	18	19	16	12	20
*A. niger CBS 513.88*	18	15	21	17	12	22

Percentage of predicted annotations for each organism across six functional descriptors, calculated from the total number of proteins lacking annotation for those descriptors. Absolute values and relative percentage to total proteome size are provided in Supplementary Data 2.

We also evaluated the accuracy of WASP in predicting existing labels for *S. cerevisiae*, a widely studied organism, and *V. natriegens*, a less conventional organism with significant annotation improvement (Table 2). For *S. cerevisiae*, the average precision across all descriptors was 0.75, while the average recall was 0.71. For *V. natriegens*, scores were substantially higher, with an average precision of 0.95 and recall of 0.87. This difference can be attributed to the higher manual curation and complex annotation performed on model organisms, which pose a greater challenge for tools to recover when compared to the more sparse annotations present for poorly characterised organisms.

Additionally, WASP outputs collateral findings, i.e. predicted annotations for organisms other than the query but enriched within the network for a certain descriptor. Of these proteins, 85% belonged to bacterial species, 12% to eukaryotes, and 2.5% to archaea. In many cases, these were less conventional and understudied species from the Alfa, Beta and Gammaproteobacteria, Actinomyces and Bacilli class in Bacteria and from the Ascomycota, Chordata, Streptophyta, Basidiomycota and Arthropoda phyla in Eukaryota.

### Full genome annotation prediction using WASP

Functional characterisation of newly sequenced genomes is a common step in bioinformatics analyses once the genome has been assembled. As a proof of concept, we employed WASP to annotate the whole genome of a novel strain, *Yarrowia lipolytica* NRRL Y-64008^43^, a new isolate of this yeast used as a biotechnological chassis for biofuels and bioproducts production. We predicted structure models for the *Yarrowia* proteome using ColabFold^44^, an optimised version of AlphaFold. Out of the 6577 open reading frames (ORFs) imputed as genes, we predicted 6575 structures. We extracted the highest ranked protein structure model and checked the overall structural models’ quality, which were found to be consistent with the scores obtained for *Yarrowia lipolytica* CLIB-122 strain retrieved from the AlphaFold Database (Supplementary Fig. 7).

WASP was run on the predicted structures using default parameters. Starting from the unannotated proteome, our analysis revealed that characterising proteins using the Pfam, PANTHER, CATH, and GO terms descriptors was more successful than identifying EC numbers and Rhea IDs for the same proteins (30% annotation vs. 10% annotations of the total proteome size, respectively Supplementary Fig. 8). The SAFE functional map shows that large clusters are linked to key cell processes like membrane transport, nucleic acid binding, metabolic and cellular processes regulation (Supplementary Fig. 9 and Supplementary Fig. 10). Additionally, we compared the functional annotations obtained through WASP with GO terms and EC number predictions from BLAST, as previously reported^43^, EggNOG and CLEAN. For CLEAN, we considered predictions with a confidence score above 0.5. Overall, EggNOG achieved the highest coverage in predicted functional annotations across the proteome. In instances where all three methods provided a prediction, there was generally high agreement among them. Moreover, WASP successfully annotated about 200 unique proteins missed by the other methods (Supplementary Fig. 11 and Supplementary Fig. 12). We investigated proteins with conflicting predictions across different methods. For instance, protein ID 22981 was identified by WASP as glucan endo-1,3-beta-D-glucosidase (EC:3.2.1.39), matching its 100% identical protein in *Y. lipolytica*, contrary to other methods identifying it as endo-1,3(4)-beta-glucanase (EC:3.2.1.6). Another protein, ID 17202, was annotated as a thiosulfate sulfurtransferase (EC:2.8.1.1) by WASP, which is supported by structural similarity to known proteins (Q08742 from *S. cerevisiae* and A0A1D8PTD8 from *C. albicans*). CLEAN predicted it as an arsenate reductase (EC:1.20.4.1), and EggNOG and BLAST did not retrieve any annotation. Combined, these examples underscore the importance of integrating structure-based tools such as WASP alongside traditional sequence-based methods to enable more comprehensive, proteome-wide annotation enriched by structural insights.

### Mapping orphan reactions to native proteins

**Fig. 4:**
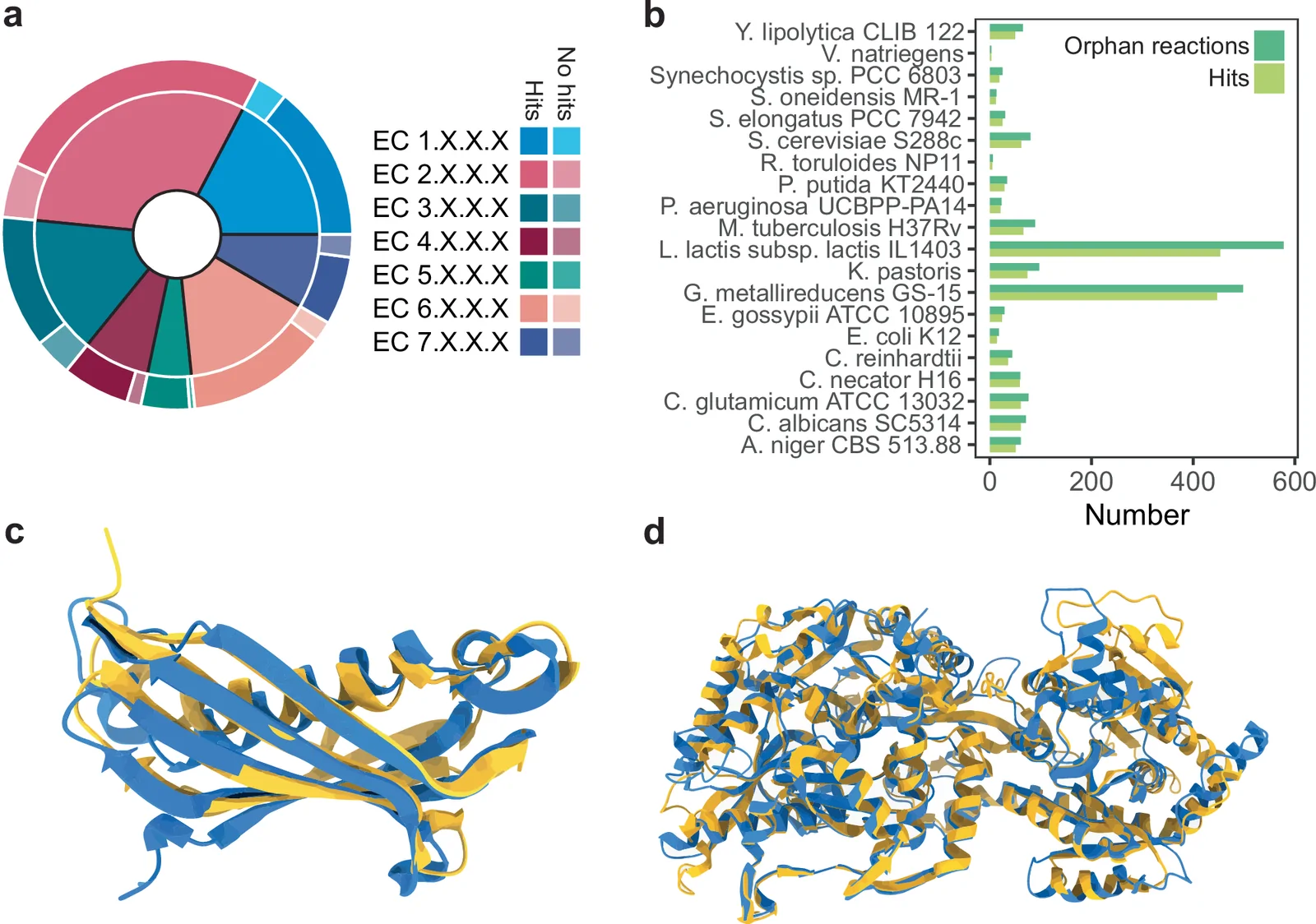
Mapping genome-scale model orphan reactions to native proteins. **a** Distribution of orphan reactions with hits across EC numbers. The inner ring represents the EC classification of the orphan reactions and the outer ring, the fraction of these annotated by WASP. **b** Distribution of orphan reactions with hits across the 20 organisms (**c**, **d**) Foldseek structural superimposition between one of the reference proteins for the orphan reaction (yellow) and its top hit in the query organism (blue) Alignments were visualised with ChimeraX ref. ^63^. In (**c**), structural homology between *Arthrobacter sp*. Q04416 and *S. cerevisiae* P38256 is shown. In (d), structural homology between *A. thaliana* B0F481 and *Y. lipolytica* Q6C529. Source data are provided as a Source Data file.

Genome-scale models (GEMs) aim to tabulate all known metabolic reactions, enabling simulation of steady-state metabolic fluxes and providing a basis to contextualise omics data and propose novel engineering strategies. Gap-filling is an essential step of GEM reconstruction, where metabolic reactions necessary for its functionality are added despite not knowing which native enzyme is performing the associated metabolic reaction (orphan reactions). Given the uncertainty surrounding the inclusion of such reactions, a major caveat in developing accurate GEMs^45^, we thus asked whether WASP could perform the reverse annotation function, that is mapping the most likely native protein to a given orphan reaction within the GEM. To test this, we used the same 20 organisms from the previous analysis (Supplementary Data 2). We retrieved the latest and most comprehensive GEM available and tailored WASP to address the specific task of refining metabolic models by gap-filling missing reactions and pathways. We first pre-processed each GEM by excluding non-enzymatic and exchange reactions (diffusion, spontaneous, biomass growth reaction and pseudo-reactions). We identified orphan reactions, which we defined as reactions present in the GEM that were not associated with any gene or enzyme. When a Rhea reaction ID or an EC number were associated with an orphan reaction, we first retrieved associated proteins from SwissProt and employed them as a reference to search for structural homologues within each of the case study organisms. We assessed the distribution of enzyme categories within orphan reactions based on the reactions they catalyse, as indicated by the first digit of their EC number, and whether we were able to map them to a reference structure in UniProt (Supplementary Table 2). A significant portion of these reactions belonged to transporter reactions (EC: 7.X.X.X) and many of these were not mapped to any reaction identifier or to proteins from other organisms. Transporters are notably much harder to map to genes due to limited gene annotation, experimental data, multi-functionality and the existence of homologous transporters with subtle substrate differences^46^. Consequently, proteins without a reference were discarded from further analysis. The set of identified proteins from SwissProt served as a database for the Foldseek alignment step against the query organism proteome. Proteins from the same database were then grouped into clusters based on their shared functional descriptor. Using the results from the structural alignment, we performed gap-filling for each orphan reaction by selecting hits in the query organism with significant alignments with all members of the corresponding cluster. WASP successfully identified hits across all EC categories (Fig. 4a) and found at least one structural match in the query organism for the majority of orphan reactions across the 20 organisms, achieving a recovery rate of 75% to 100% (Fig. 4b). Most of these hits exhibited high average TM-scores (>0.7), indicating strong structural homology, and are currently poorly annotated in UniProt, with an average annotation score of less than 2 (out of 5) across all hits per organism.

Predicted annotations for orphan reactions were identified in well-annotated organisms, including *S. cerevisiae*. For example, P38256, currently uncharacterised in the reference *Saccharomyces* Genome Database (SGD)^47^ is ambiguous according to Uniprot, indicated both as a probable thioesterase (EC:3.1.2.-) or as part of the acyltransferase family (EC:2.3.1.-). WASP identified it as the best scoring hit for the orphan reaction 4-hydroxybenzoate formation converting 4-hydroxybenzoyl-CoA to 4-hydroxybenzoate (EC: 3.1.2.23). WASP associated it with two thioesterases: Q04416 from *Arthrobacter* sp., and P56653 from *Pseudomonas* sp. (strain CBS-3). Despite a high TM-score (0.72) with the two proteins, P56653 only had 12% sequence identity, a value well below the usual threshold used to infer homology through sequence, explaining the low annotation score (Fig. 4c).

Another example is the orphan reaction dethiobiotin synthase (EC: 6.3.3.3) in *Yarrowia lipolytica*. WASP identified Q6C529 as the most likely native enzyme performing this reaction. This protein is reported as uncharacterised in UniProt, with a low annotation score of 2 out of 5, and is suggested to be involved in biotin synthesis. Q6C529 is structurally very similar to 3 proteins annotated in SwissProt as dethiobiotin synthases, B0F481 from *A. thaliana*, Q6ZKV8 from *O. sativa*, and P13000 from *E. coli*, with a TM-score ranging from 0.74 (bacterial) to 0.84 (plant). Sequence similarity, however, is low (32% and 21% homology with the plant and bacterial enzymes, respectively), again explaining the low annotation score (Fig. 4d). B0F481 and Q6ZKV8 are bifunctional proteins with distinct domains for ATP-dependent dethiobiotin synthetase and 7,8-diaminopelargonic acid (DAPA) aminotransferase, both of which align with the *Yarrowia* candidate. In contrast, the bacterial P13000 is an ATP-dependent dethiobiotin synthetase, with only one domain aligning to the *Yarrowia* protein, explaining the lower alignment score. This suggests that Q6C529 can perform both functions, similar to the bifunctional enzymes in other species. Other Saccharomycotina species, including *S. cerevisiae*, possess two separate genes (*BIO3* and *BIO6*), oriented in opposite directions, encoding the two enzymes separately. Previous studies suggest that the biotin biosynthetic pathway was lost in an ancestor of *S. cerevisiae* and other hemiascomycetes, and later reacquired through separate horizontal gene transfer from bacteria and gene duplication events. Instead, evidence of single bifunctional proteins is observed in various species of basidiomycetes, filamentous fungi, and *Yarrowia*^48^. To further investigate the distribution of biotin gene cluster across the Dikarya subkingdom, we decided to use a structure-based approach. Using Foldseek, we identified homologues (TMscore > 0.75) of the *Yarrowia* protein Q6C529 across AlphaFold DB, setting a query coverage of 0.8 to exclude single domain proteins. We then performed a similar search for the *S. cerevisiae* protein P50277, encoding the DAPA aminotransferase, using a target coverage of 0.8 to exclude bifunctional enzymes. This resulted in around 20 proteins for each gene organisation type across Dikarya. The resulting phylogenetic tree demonstrates the irregular distribution of the biotin gene cluster, with closely related organisms displaying different organisations (Fig S13). This suggests a novel perspective on evolutionary studies, showcasing the potential of using a structure-based approach in cases where gene organisation and function exhibit significant variability.

### WASP reconciles in silico essentiality prediction with loss-of-function screens

To further demonstrate the biological utility of WASP, we integrated its predicted protein hits into the GEMs of three pathogenic organisms—*Helicobacter pylori*, *Pseudomonas aeruginosa*, and *Mycobacterium tuberculosis*—for which high-quality transposon mutant libraries are available. We assessed GEM model performance before and after WASP-based refinement by comparing simulated gene essentiality predictions with experimental data under matched growth conditions. GEM accuracy was evaluated by calculating true positives (TP), false positives (FP), false negatives (FN), and true negatives (TN), and computing precision, recall, and F1 scores. In addition to expanding gene-reaction associations, we used WASP to address false positives—genes incorrectly predicted as essential in silico but known to be non-essential experimentally. Such misclassifications often stem from missing annotations of redundant homologues capable of compensating for gene knockouts. WASP was used to identify structural homologues of these FP genes or of known enzymes catalysing the same reaction in other organisms. These were added to the GEMs using updated gene–protein–reaction (GPR) rules with logical OR relationships, and essentiality simulations were re-evaluated.

In *H. pylori*, this refinement increased precision from 0.56 to 0.59, and in *P. aeruginosa*, from 0.49 to 0.58. In both organisms, many of the identified genes had low UniProt annotation scores (<3), highlighting WASP’s value in recovering biologically relevant functions from under-annotated regions.

The most substantial improvements were observed in *M. tuberculosis*, where precision increased from 0.82 to 0.92 and the F1 score from 0.77 to 0.81. Several of the mapped reactions corresponded to proteins experimentally confirmed as essential or listed in UniProt as high-potential drug targets. For instance, WASP linked Rv1202 to the reaction acetylornithine deacetylase (ArgE), an enzyme absent in humans and thus a promising antibiotic target. While UniProt annotates Rv1202 as a putative succinyl-diaminopimelate desuccinylase (DapE), it belongs to the ArgE/DapE metallohydrolase family, and previous studies have noted the structural and mechanistic similarities between these homologues. Both enzymes have been explored as antibacterial targets, and inhibitors developed against DapE often show cross-reactivity with ArgE^49^. WASP’s assignment suggests that Rv1202, though annotated as DapE-like, may also participate in arginine metabolism, or at minimum, shares druggable features with ArgE. In addition, WASP identified Rv2141c as a second candidate for the same reaction, with comparable structural similarity and domain architecture. Importantly, Rv2141c has been implicated in in vivo virulence, underscoring its relevance during host infection even if not required for in vitro growth. This raises the possibility of functional redundancy within this enzyme family in *M. tuberculosis*, which has not been systematically characterised. By highlighting both Rv1202 and Rv2141c as plausible contributors to this reaction, WASP reveals an expanded and potentially more flexible metabolic landscape that could be exploited for multi-target inhibition strategies that consider both in silico essentiality and in vivo survival requirements.

Another case involves Rv2172c, an unreviewed conserved protein in UniProt, which WASP functionally mapped to 5,10-methylenetetrahydrofolate reductase (MTHFR)—a key reaction in folate and one-carbon metabolism. Although Rv2172c had not been linked to this specific reaction in GEMs, it shares structural domains with MTHFRs characterised in *M. smegmatis*. These bacterial enzymes are distinct in being NADH-dependent and flavin-independent—unlike their human counterparts—offering a potential selectivity window for inhibition^50^. Supporting this idea, a follow-up study identified small molecule inhibitors, such as compound AB131, that bind the NADH pocket of MsmMTHFR, suggesting Rv2172c may be similarly druggable^51^ (Fig. S14).

Finally, WASP resolved several false positives in the mycolic acid biosynthesis pathway, a subsystem known for its complexity, essentiality, and role in drug resistance and virulence^52,53^. By identifying previously overlooked homologues and refining the logic of gene–reaction mappings, WASP helped distinguish between compensatory and functionally essential enzymes, enhancing GEM fidelity and highlighting more specific points of intervention. Together, these results demonstrate how WASP enhances GEM accuracy, provides mechanistic clarification, and enables translational insights from proteins that are partially or ambiguously annotated in current databases.

## Discussion

In this study, we introduce WASP, a structure-based tool for protein functional annotation prediction derived from AlphaFold structural models. Compared to traditional sequence-based methods, WASP takes advantage of the intrinsic connection between a protein 3D shape and its molecular activity, providing more accurate and reliable predictions even for proteins with distant evolutionary relationships, shown here with the FtsZ/tubulin family. WASP’s network-based representation of structural homology also provides robustness during annotation, where neighbour connectivity and local graph topology provide the statistical power for the creation of a proteome functional map. This topology is leveraged by identifying functionally coherent clusters and propagating information beyond direct hits, enabling annotation based on a protein’s extended local environment in the graph. The main caveat with this approach is that a putative annotation is as good as its neighbouring annotations. Absence of annotations can give rise to dark clusters, i.e. disconnected subnetworks where no member possesses any form of annotation. These include poorly predicted proteins with low-confidence structural models or with uncommon and underrepresented folding architectures. Addressing these dark clusters is crucial, as they represent genuine gaps in our knowledge, and WASP could thus be used to prioritise future experimental validation.

Our analysis specifically focused on WASP’s ability to identify EC numbers, the most detailed functional annotation. There, WASP demonstrated superior precision and recall when compared to current machine-learning based tools, including CLEAN, especially for better characterised enzymes such as those in the New-392 dataset. In the more challenging Price-149 dataset, WASP produced complementary results to CLEAN, resulting in an overall higher precision and recall when both tools were combined. In fact, for the same dataset, the combined tools achieved the same scores as a contrastive learning tool trained on a set of proprietary sequences from diverse and understudied environments^54^ (Supplementary Fig. 6).

Most proteins misannotated by WASP in Price-149 differed from the true labels only by the fourth digit of the EC number, which denotes substrate preferences. In contrast, WASP achieved better scores than CLEAN when considering the more generic third digit. In fact, enzymes are often able to recognise and accept different substrates, a phenomenon referred to as promiscuity^55^. As such, another explanation for WASP’s difficulty to predict the fourth digit could also indicate enzymes with promiscuous activities. Alternative approaches for enzyme classification have started to emerge, focusing on more generic reaction rules to indicate the underground metabolism caused by enzyme promiscuity^56^. We found that about one-fourth of the proteins misannotated by WASP had predicted labels that followed the same reaction rules as the true labels (Supplementary Data 4), suggesting WASP utility in identifying multiple activities from structural homology and implying that accounting for enzyme promiscuity could further improve the annotation accuracy.

We also showed WASP utility for applied research, it could standardise annotation across model and non-conventional organisms that are emerging as promising chassis for biotechnological use yet remain poorly characterised. For instance, *Y. lipolytica* has gained interest in both academia and industry for its unique metabolic, genetic and physiological features. It has been widely used in food applications, chemicals and biofuels production and bioremediation^57^. Similarly, *V. natriegens* has emerged as a promising next-generation chassis for recombinant protein production thanks to its rapid growth rate and substrate utilisation versatility^58^.

WASP also builds on previous tools aimed at improving GEMs by addressing metabolic gaps. Earlier methods focused on reconciling discrepancies where GEMs predicted essential reactions not supported by experimental data, by proposing alternative reactions, assessing their thermodynamic feasibility, and suggesting candidate genes^59^. More recently, Graph Convolutional Networks (GCNs) have been used to represent the metabolic network, learn its topological features to identify gaps and propose specific reactions that make the network more complete and functionally coherent^60^. WASP tackles the critical problem of missing gene-reaction associations by employing structural homology. By linking orphan reactions to native proteins through structural alignment, WASP ensures these reactions are grounded in plausible biological associations. Further analysis of missing enzymes could also unravel horizontal gene transfers such as members of *S. cerevisiae* biotin synthesis acquired from bacteria. In fact, pan-proteome classification based on structure could provide insights on phylogenetic trees and evolution. We note for instance that WASP predicted annotation of whole proteomes was a better clade discriminator than when based on sequence (Supplementary Note 1).

Together, our results stress the need to incorporate structural information into the next generation of protein annotation prediction tools. The improved annotations provided in this study combined with WASP’s ease of use will serve as a valuable resource for computational approaches and experimental techniques, contributing to the development of innovative biotechnological applications and more targeted bioengineering strategies. Another potential area for improvement is the structural alignment component of our pipeline, which is currently performed by Foldseek. It is important to note that Foldseek does not incorporate prior knowledge during the alignment process. Consequently, critical functional motifs, such as those surrounding the catalytic site, are weighted similarly to regions less critical to enzyme function. Incorporating functional-site awareness, as explored in PARSE^61^, could further enhance annotation accuracy. As structural prediction technologies continue to evolve, tools like WASP will be instrumental in unlocking the full potential of proteomic data, driving advancements in fundamental research, applied biotechnology and synthetic biology. Together with machine-learning based tools, structural homology holds great promise for superior functional annotations.

## Methods

### WASP implementation details

WASP is a python-based command line tool designed to annotate whole-proteomes based on structural homology. The pipeline requires Foldseek and gsutil from Google Cloud services to be pre-installed. The full code, required python packages and usage instructions are provided at https://github.com/gioodm/wasp-proteins-annotation. All study data are included in the article and/or supporting information. The software is also permanently archived on Zenodo with https://doi.org/10.5281/zenodo.20617135 ref. ^62^. Source data for all Figures in the main manuscript and in the Supplementary Information are provided with this paper.

WASP comprises four main steps:

1. Data retrieval: AlphaFold structural models for the query organism defined on input through its corresponding NCBI taxonomy ID are downloaded from the AlphaFold Database Google Cloud Bucket through the gsutil tool. Reference protein structures were obtained from the AlphaFold Protein Structure Database (AFDB, version 4, accessed September 2024). Where applicable, the model’s unique UniProt accession are provided in the source data. Even when experimentally resolved structures are available in the PDB, WASP uses AlphaFold-predicted models to ensure consistency across the entire proteome. Foldseek is used to retrieve, index and combine SwissProt and the AlphaFold Structure Database clustered at 50% sequence identity and 90% bidirectional coverage.

2. Structural alignment: Two Foldseek alignments are performed. The search is first executed all-against-all in one direction, wherein the query organism structural models are searched against the combined databases described above, as well as the query organism proteome itself (foldseek easy-search, standard parameters). The predicted structural models for the query organism are included in the database to account for possible paralogs. Subsequently, the highest scoring hit for each query organism protein is retained as its best hit, using default significance thresholds of bitscore > 50 and e-value < 1e-10. To increase confidence in the structural matches, the retained best hits are then structurally blasted against the same database to obtain the reciprocal hits (using Foldseek createsubdb, search, and convertalis). Using the Reciprocal Best Hit (RBH) strategy, pairs of proteins are retained only when a significant alignment (bitscore > 50 and e-value < 1e-10) is found in both directions — that is, the query protein must be among the top hits (i.e., in the neighbourhood) of its best hit, and vice versa. By default, each neighbourhood is defined as the top 10 matches per protein. All parameters, including neighbourhood size and significance thresholds, are customisable by the user. To enable users to focus on distant evolutionary relationships, users can also exclude potential sequence homologues by defining a sequence identity threshold; hits exceeding this percentage are filtered out prior to network generation and functional enrichment.

3. Network construction: The protein pairs from the previous step are used to build an undirected weighted network using Python3 networkx package. Nodes represent protein pairs and edges are weighted based on the alignment bitscore, normalised between 0 and 1. Clusters in the obtained network are sorted by size (number of nodes) and reported as output, together with their average degree and clustering coefficient metrics.

4. Functional Inference: Current known annotation for each protein in the network is retrieved from UniProt REST API. Functional annotation is defined through six descriptors: Pfam and CATH domains, PANTHER subfamily classification, Enzyme Commission (EC) number, Rhea reaction ID and Gene Ontology (GO) terms.

Enrichment analysis is performed for each disconnected cluster in the network using the Spatial Analysis of Functional Inference (SAFE) pipeline^37^. For each of the six descriptors, SAFE defines local neighbourhoods for each node. Neighbourhoods are indicated as a set of nodes located within a certain distance but not necessarily directly connected. For each neighbourhood, SAFE calculates a quantitative score corresponding to the sum of neighbours’ values for a chosen functional attribute, in this case represented by the previously retrieved annotation labels. Enrichment is performed using a one-tailed Fisher’s exact hypergeometric test, comparing the observed functional attribute values to random. P-values are corrected for multiple testing, filtered by significance (p-value < 0.05) and log-transformed into neighbourhood enrichment scores. Inferred labels and their neighbourhood scores are then assigned to corresponding nodes on the network map and reported as output. Total counts of predicted annotations for each descriptor are reported as barcharts, subdivided into proteins from the query organism and proteins from other organisms (collateral findings). The network construction and enrichment analysis steps are repeated for *n* iterations (default = 3) with progressively relaxed parameters, by increasing the number of hits for each query included in the initial network (by default, the initial network includes 10 neighbours per query and the number is increased by 10 with each subsequent iteration). This can help in achieving additional annotation for proteins that still have no annotation in two ways: 1. linking clusters that were before disconnected by including more neighbours that can bridge the gap; 2. including annotated proteins in the network that allow reaching significant scores during enrichment.

### WASP benchmark

#### *E. coli* and *S. cerevisiae* benchmarking

We selected two well-characterised organisms, *Saccharomyces cerevisiae S288c* and *Escherichia coli K12*, as queries. For each of them, we retrieved all proteins in UniProt that were annotated for all the six functional descriptors used by WASP (1108 and 1142 proteins respectively) and used these collections as test data. We executed WASP with default parameters, hiding the correct labels for these proteins during the annotation retrieval step. Evaluation metrics included precision, recall and F1 scores and were calculated using Python3 scikit-learn library. First, ground truth and predicted labels were binarized; then these were used as input to compute the metrics, according to scikit-learn guidelines. A weighted average was employed to account for the multi-label setting. We also evaluated the impact of using multiple iterations of the network construction-functional inference steps: 10 iterations were executed with increasing number of hits included in the network (from 10 to 100, with step 10).

#### Sequence-based WASP

The sequence-based version of WASP was obtained by substituting Foldseek with the sequence alignment tool MMsesq2. We created a sequence-based version of the AlphaFold DB clustered at 50% identity and adapted MMseqs2 default search parameters to match the ones used by Foldseek, by setting the maximum results per query sequence allowed to pass the prefilter to 1000 and sensitivity to 9.5. The rest of the pipeline was not changed.

#### WASP performance against other annotation tools

WASP was benchmarked against four sequence-based annotation tools: CLEAN, ProteInfer, DeepEC and EggNOG-mapper. For CLEAN, pre-trained weights were taken out-of-the-box directly from its GitHub repository (https://github.com/tttianhao/CLEAN). The latest version of EggNOG was installed following GitHub instructions (https://github.com/eggnogdb/eggnog-mapper). DeepEC was installed using its source code available at https://bitbucket.org/kaistsystemsbiology/deepec/src/master/. Scores for ProteInfer were obtained from ref. ^7^ due to constraints in creating a free GPU instance with sufficient resources on Google Cloud Platform, as specified in the tool usage instructions at https://github.com/google-research/proteinfer. Two test dataset – New-392 and Price-149 – from ref. ^7^ were used to compare the methods. New-392 comprises 392 enzymes covering 177 EC numbers, with data sourced from SwissProt released after CLEAN was trained (April 2022). Price-149 is a set of experimentally validated results. As before, labels were hidden during the WASP annotation step. 10 total iterations were executed with an increasing number of hits. Precision, recall and F1 scores were calculated with Python3 scikit-learn library as described before.

#### Whole-proteome annotation

WASP was run on a set of 20 industrially relevant organisms, including bacteria, yeast and fungi (Supplementary Data 2) with default parameters.

The optimised version of AlphaFold, ColabFold^44^, was used from terminal to predict structure models for the *Yarrowia lipolytica* NRRL Y-64008^43^ proteome using a NVIDIA GPU on the Imperial College high-performance cluster facility. ColabFold was run using default parameters and the template and amber relaxation flags. Full-length amino acid sequences for all Y. lipolytica proteins were used as inputs to ColabFold without any truncation or modification. Out of the 6577 open reading frames (ORFs) imputed as genes, we predicted 6575 structures, while the remaining two proteins (with amino acid residues > 4500) were excluded due to hardware limitations. The highest ranked prediction protein structural models were extracted and used for proteome annotation using WASP. Protein structural models quality checks were performed, including average predicted Template Modelling score (pTM) and average predicted Local Distance Difference Test (pLDDT).

#### Genome-scale model gap-filling adaptation

The WASP workflow was partially adapted to address the genome gap-filling problem in GEMs. For each of the 20 organisms above the most recent and/or complete GEM model was retrieved (Supplementary Data 2).

The main differences with the general WASP workflow are: 1. The GEM model files (accepted extensions:.xml,.sbml,.json,.mat) were pre-processed to create standardised input files for WASP, which need to be supplied together with the organism taxonomy ID. These steps were performed manually and are not integrated into the automated pipeline workflow; code is provided but might require modifications depending on the GEM format. During this step, orphan reactions – defined as reactions that are not associated with any gene or enzyme and are not exchange reactions – are identified using Python3 cobrapy package (find_orphans.py). Non-enzymatic (diffusion, spontaneous, biomass growth and pseudo-reactions) reactions were excluded from the analysis. Each of the remaining reactions is mapped to the corresponding Rhea reaction ID and/or EC number, when available (rxn2code.py). The resulting output file includes each orphan reaction alongside any associated Rhea ID/EC number.

2. The structural BLAST step with Foldseek is executed only against an in-house database of proteins from SwissProt associated with the same catalytic functions as the ones found to be orphan in the organism GEM. These proteins are identified using their associated Rhea ID/EC number. Proteins are filtered to include only the ones with experimental validation and an AlphaFold-predicted structure.

3. To ensure high-quality results, the alignment results by Foldseek are also filtered by TM-score, a measure of structural similarity. A threshold value of 0.5 was chosen as it is generally considered as threshold for orthology.

4. Clusters are obtained using proteins from the in-house database sharing the same enzymatic function. To account for possible divergent structures in proteins performing the same activity, each possible pair in the cluster is also structurally aligned and a graph is created to identify subclusters of structurally similar proteins within them (TM-score > 0.5). Gap-filling is performed for each subcluster separately, taking as best alignment hits only significant hits in the query organism shared by all proteins in the subcluster. Best hits are then ordered by average TM-score and reported as output.

#### Integration of WASP predictions into GEMs for essentiality evaluation

For each of the three test organisms (*H. pylori*, *P. aeruginosa*, and *M. tuberculosis*), we selected one high-quality transposon mutant library from published studies and used these datasets as experimental ground truth for gene essentiality. GEM simulations were performed in Python3 using COBRApy, and growth media for each organism were adapted to match, as closely as possible, the experimental conditions described in the corresponding transposon study. WASP predictions were incorporated into GEMs in two contexts. For orphan reactions, top WASP hits were added as gene–protein–reaction (GPR) associations. For false positives (genes predicted as essential in silico but experimentally non-essential), WASP-identified homologues were introduced into the existing GPRs as logical OR relationships specifically for the gene misclassified as essential. For example, if the original GPR was *gene1 and gene2* and WASP identified *gene3* as a structural homologue of the false positive *gene1*, the updated GPR became (*gene1 or gene3*) and *gene2*. GEM model performance before and after WASP refinement was evaluated by comparing predicted essentiality against transposon datasets, with precision, recall, and F1 scores calculated from true positives (TP), false positives (FP), true negatives (TN), and false negatives (FN).

#### Cross-organism comparison using WASP annotation

WASP was executed on an additional set of 14 organisms using standard parameters. For each of the six functional descriptors, two binary matrices were generated: one representing pre-existing annotations from UniProt and the other incorporating annotations obtained through WASP. In these matrices, annotation labels served as features, while rows represented the organisms. A value of 1 indicated the presence of at least one protein annotated with the corresponding feature in the organism, whereas a value of 0 indicated its absence. The matrices were filtered to remove highly correlated features as well as features that were constant or uniform in more than 90% of the organisms. PCA analysis was conducted on the filtered matrices. Hierarchical agglomerative clustering was performed on the PCA results, using Euclidean distance and the average linkage method. Clustering information distance was calculated using the TreeDist package in R, allowing comparison of each tree to a reference NCBI tree. The interactive Tree of Life (iTOL) online tool was used to visualise the results.

### Reporting summary

Further information on research design is available in the Nature Portfolio Reporting Summary linked to this article.

## Supplementary information


Supplementary Information
Description of Additional Supplementary Files
Supplementary Data 1
Supplementary Data 2
Supplementary Data 3
Supplementary Data 4
Reporting Summary
Transparent Peer Review file


## Source data


Source Data


## Data Availability

Unless otherwise stated, all data supporting the results of this study can be found in the article, supplementary, and source data files. The AlphaFold structural models used for reference were accessed from the AlphaFold Protein Structure Database [https://alphafold.ebi.ac.uk/] (version 4, accessed September 2024). The predicted structural models for Yarrowia lipolytica NRRL Y-64008 generated with ColabFold have been deposited in Zenodo [https://doi.org/10.5281/zenodo.20715958] and are available under the Creative Commons Attribution 4.0 International (CC BY 4.0) license. Benchmark validation datasets are available through the project’s GitHub Release platform [https://github.com/gioodm/wasp-proteins-annotation]. The software is also permanently archived on Zenodo [https://doi.org/10.5281/zenodo.20617135]. Source data for all Figures in the main manuscript and in the Supplementary Information are provided with this paper. Source data are provided with this paper.

## Code availability

The pipeline software code and detailed deployment instructions are available in the project’s GitHub repository [https://github.com/gioodm/wasp-proteins-annotation]. The production version used in this study is permanently archived in Zenodo [https://doi.org/10.5281/zenodo.20617135] under the MIT License.
