# Supplementary material for: WASP: a pipeline for functional annotation prediction based on AlphaFold structural models
Source: Nat Commun. 2026 Jul 28;17:9156. doi: 10.1038/s41467-026-75956-z (PMC13522567; doi:10.1038/s41467-026-75956-z)
Supplement: Supplementary file 1 [file 41467_2026_75956_MOESM1_ESM.pdf]

Supplementary Information

WASP: A pipeline for functional annotation prediction based on AlphaFold structural models

Giorgia Del Missier, Kiyan Shabestary, Rodrigo Ledesma-Amaro

Supplementary Figures

Supplementary Figure 1. Sequence and structure alignment scores comparison in the FtsZ/tubulin protein family.
Supplementary Figure 2. Precision and recall scores in the *E. coli* dataset.
Supplementary Figure 3. Precision and recall scores in the *S. cerevisiae* dataset.
Supplementary Figure 4. Sequence and structure alignment scores comparison of best first hits in the *E. coli* and *S. cerevisiae* datasets.
Supplementary Figure 5. Comparative Venn diagrams of classification accuracy by WASP and CLEAN in the New-392 and Price-149 datasets.
Supplementary Figure 6. Additional benchmark scores on Price-149 dataset.
Supplementary Figure 7. *Y. lipolytica* NRRL Y-64008, AlphaFold models' quality checks.
Supplementary Figure 8. *Y. lipolytica* NRRL Y-64008, annotation statistics.
Supplementary Figure 9. *Y. lipolytica* NRRL Y-64008, SAFE functional map.
Supplementary Figure 10. *Y. lipolytica* NRRL Y-64008, enrichment dotplot.
Supplementary Figure 11. *Y. lipolytica* NRRL Y-64008, GO terms upset plot.
Supplementary Figure 12. *Y. lipolytica* NRRL Y-64008, EC number upset plots.
Supplementary Figure 13. Biotin gene cluster organisation across the Dikarya subkingdom.
Supplementary Figure 14. Molecular docking of *M. tuberculosis* Rv2172c with AB131.

Supplementary Tables

Supplementary Table 1. Examples for WASP augmented annotations in two model organisms.
Supplementary Table 2. Enzyme categories distribution within orphan reactions.

Supplementary Note

Supplementary Note 1. WASP improves phylogenetic tree reconstruction using phenotypic annotation.

Supplementary Data

Supplementary Data 1. Comprehensive list of GTPase FtsZ/Tubulin protein family (CATH Superfamily 3.40.50.1440) proteins from SwissProt used in analysis. (.xlsx)
Supplementary Data 2. The cohort of 20 model organisms relevant for industrial applications used in this study and links to the corresponding GEM used for curation. (.xlsx)
Supplementary Data 3. Complete annotation results statistics for the 20 organisms as determined by WASP. (.xlsx)
Supplementary Data 4. WASP prediction vs true labels in the Price-149 dataset. Predictions following common reaction rules with the true labels are highlighted. (.xlsx)

Supplementary Figure 1. Comparison of sequence and structure alignment scores between cellular division protein FtsZ and Tubulin beta-chain pairs from SwissProt, with predicted structure available in the AlphaFold Database. Sequence alignment scores were obtained using MMseqs2, while structural alignment scores were derived using Foldseek. Pairs of proteins that did not produce alignment outputs were assigned a score of 0.

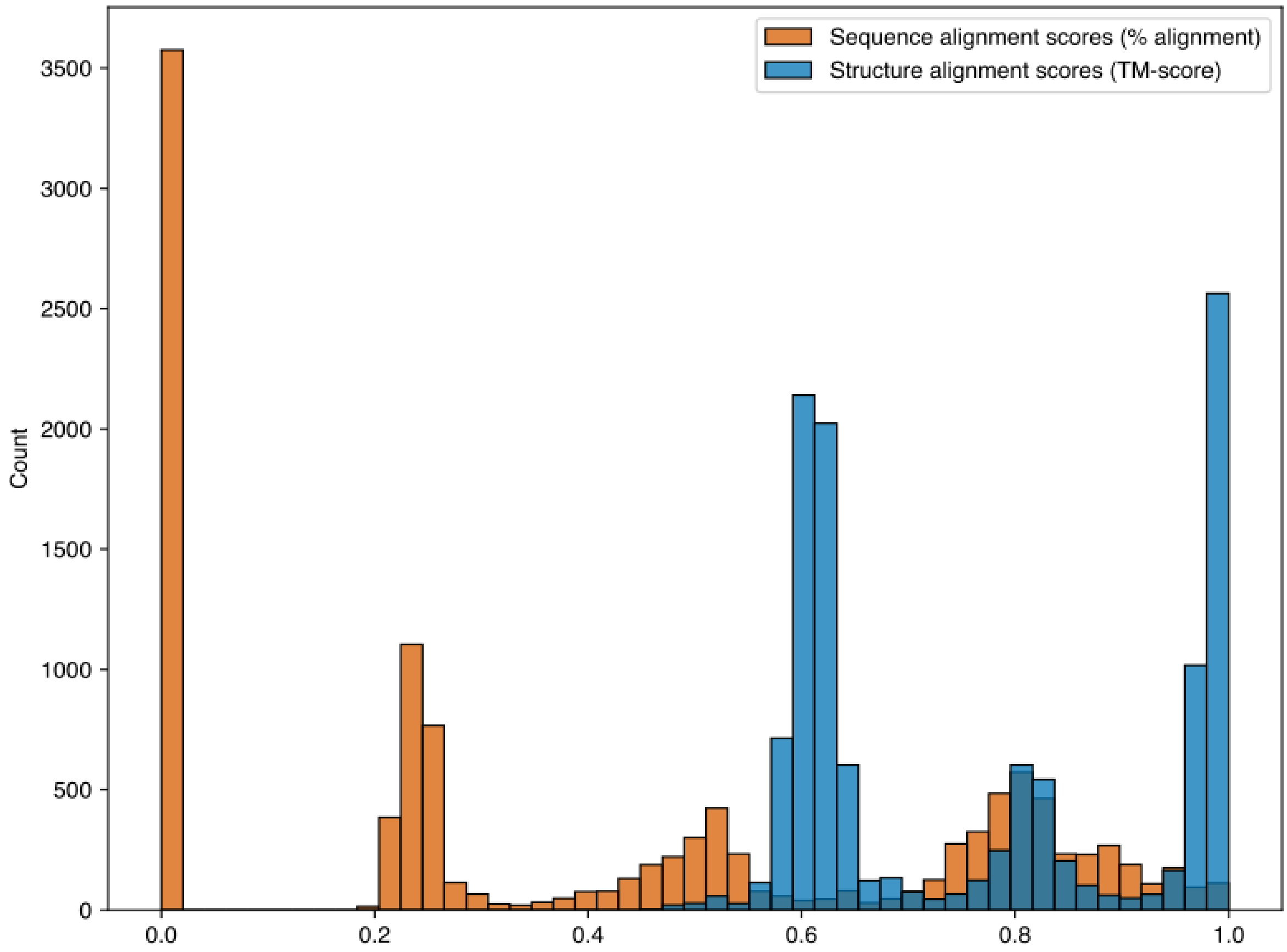

Supplementary Figure 2. Precision and recall scores across iterations for each of the six functional descriptors considered in the study in the *E. coli* dataset, comprising 1142 well-characterised proteins.

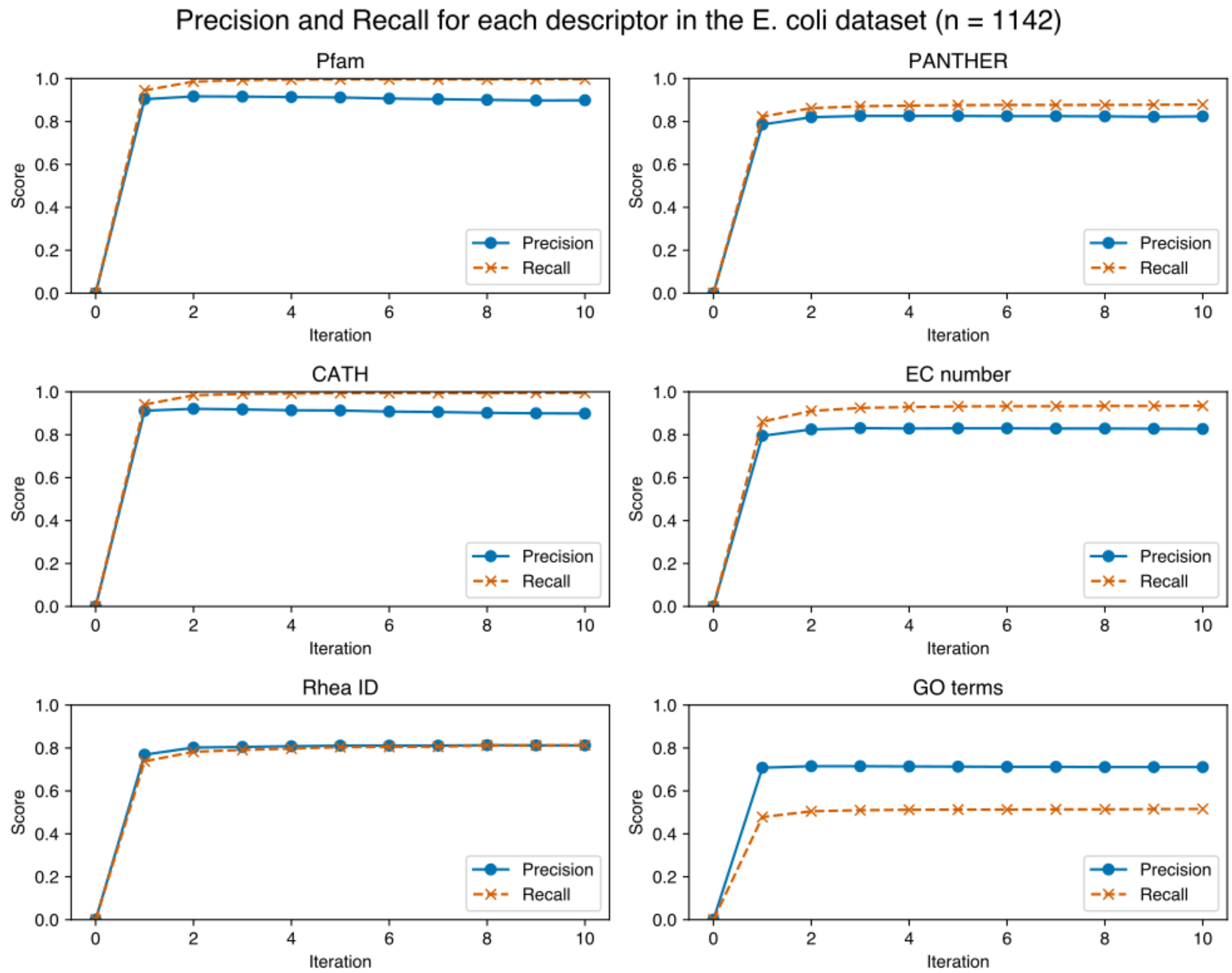

Supplementary Figure 3. Precision and recall scores across iterations for each of the six functional descriptors considered in the study in the *S. cerevisiae* dataset, comprising 1108 well-characterised proteins.

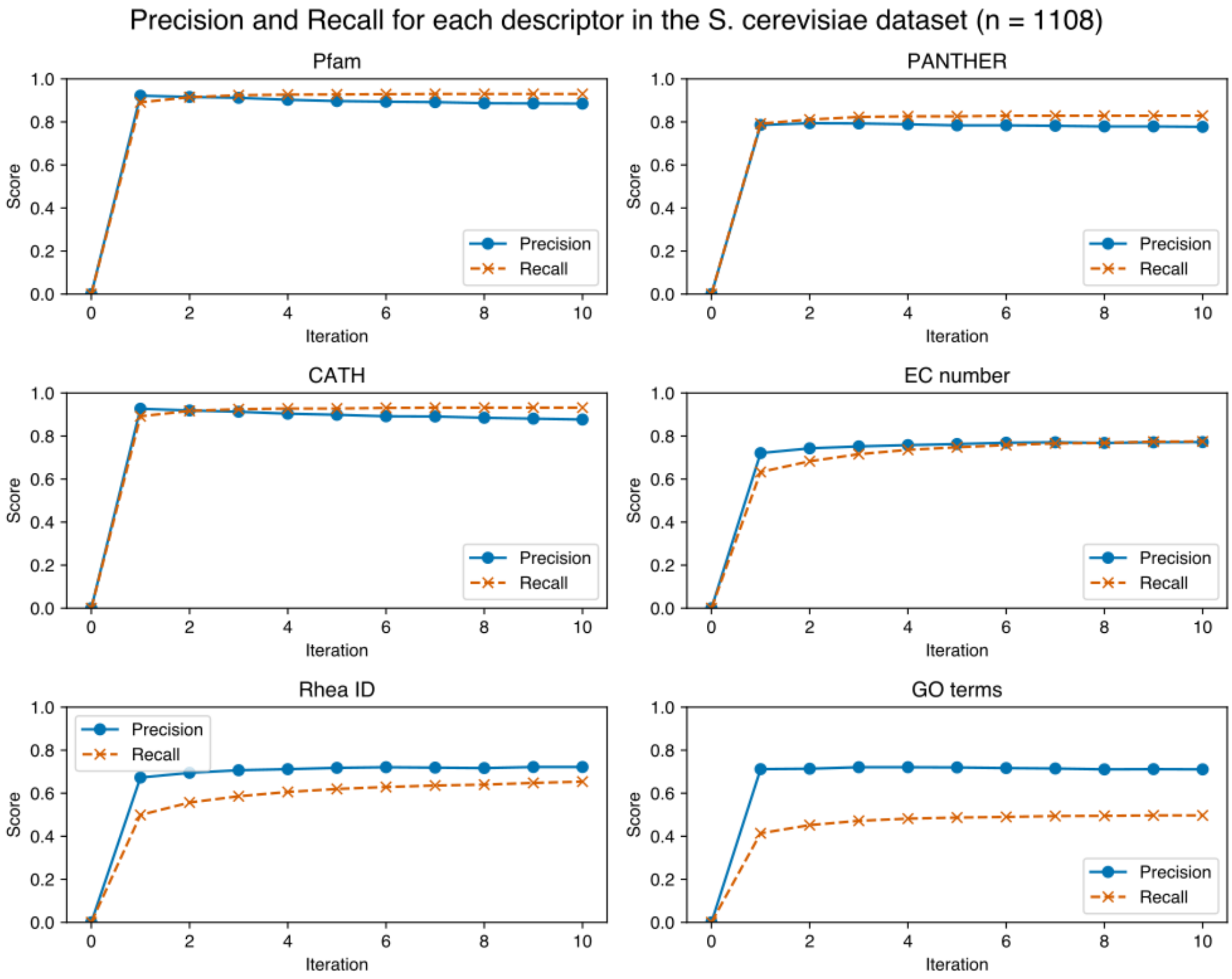

Supplementary Figure 4. Kernel density plot fitted on the sequence and structure alignment scores between proteins and their highest-scoring structural hit identified using Foldseek in the *E. coli* (**a**) and *S. cerevisiae* dataset (**b**).

a.

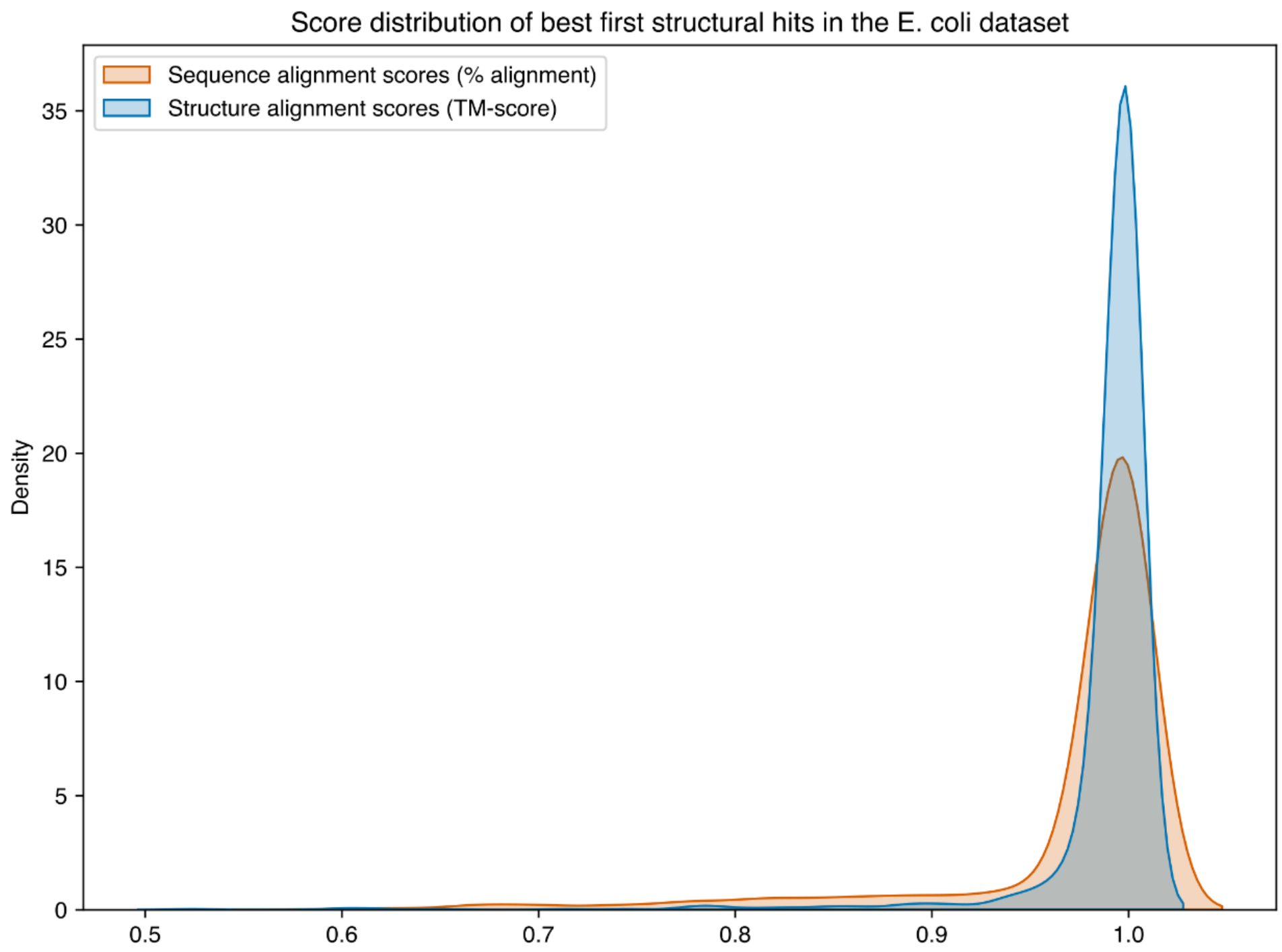

b.

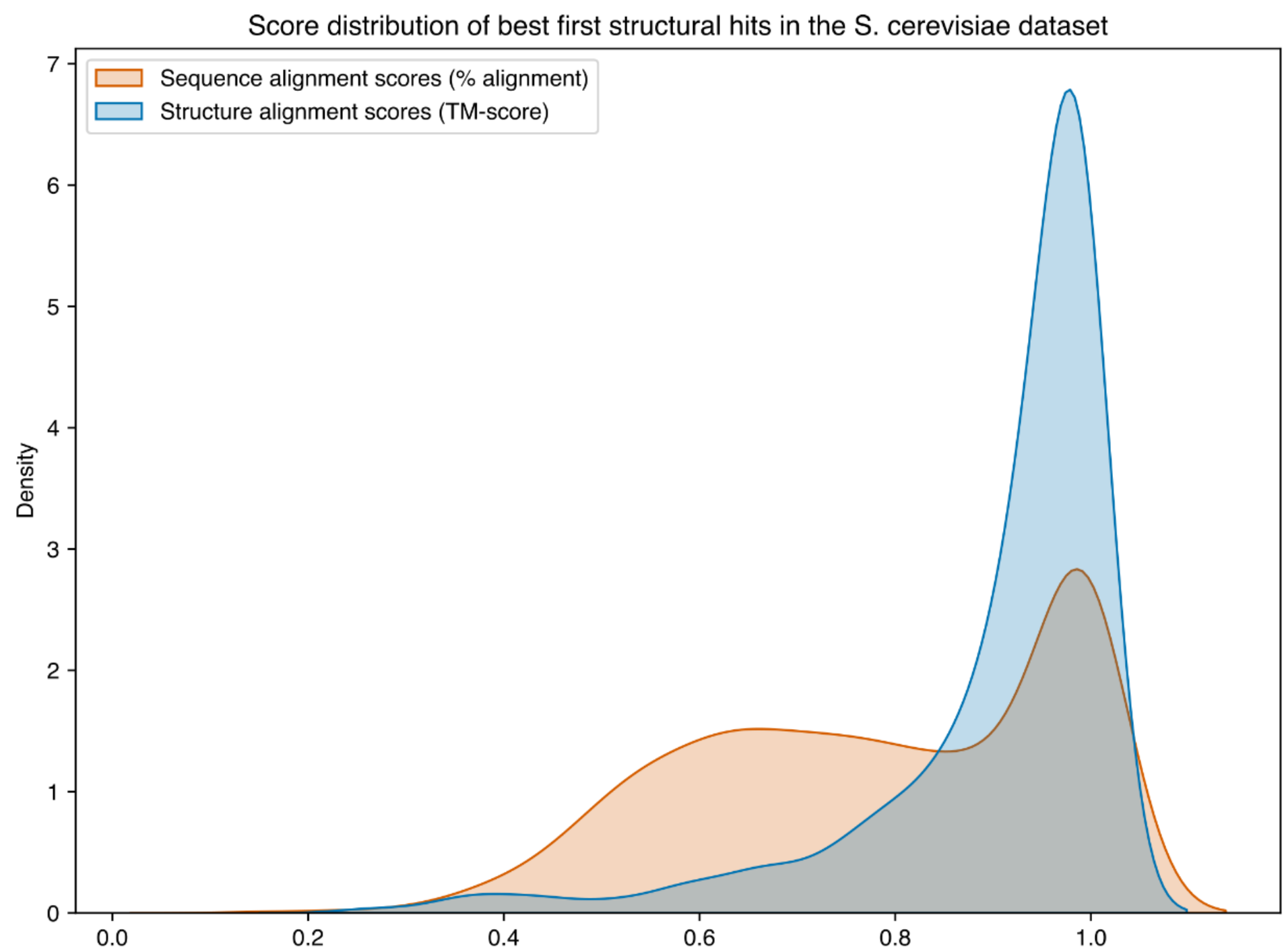

Supplementary Figure 5. Comparative Venn diagrams showcasing the overlap of correctly classified instances by two different methods, WASP and CLEAN, in the New-392 (**a**) and Price-149 (**b**) datasets.

a. New-392

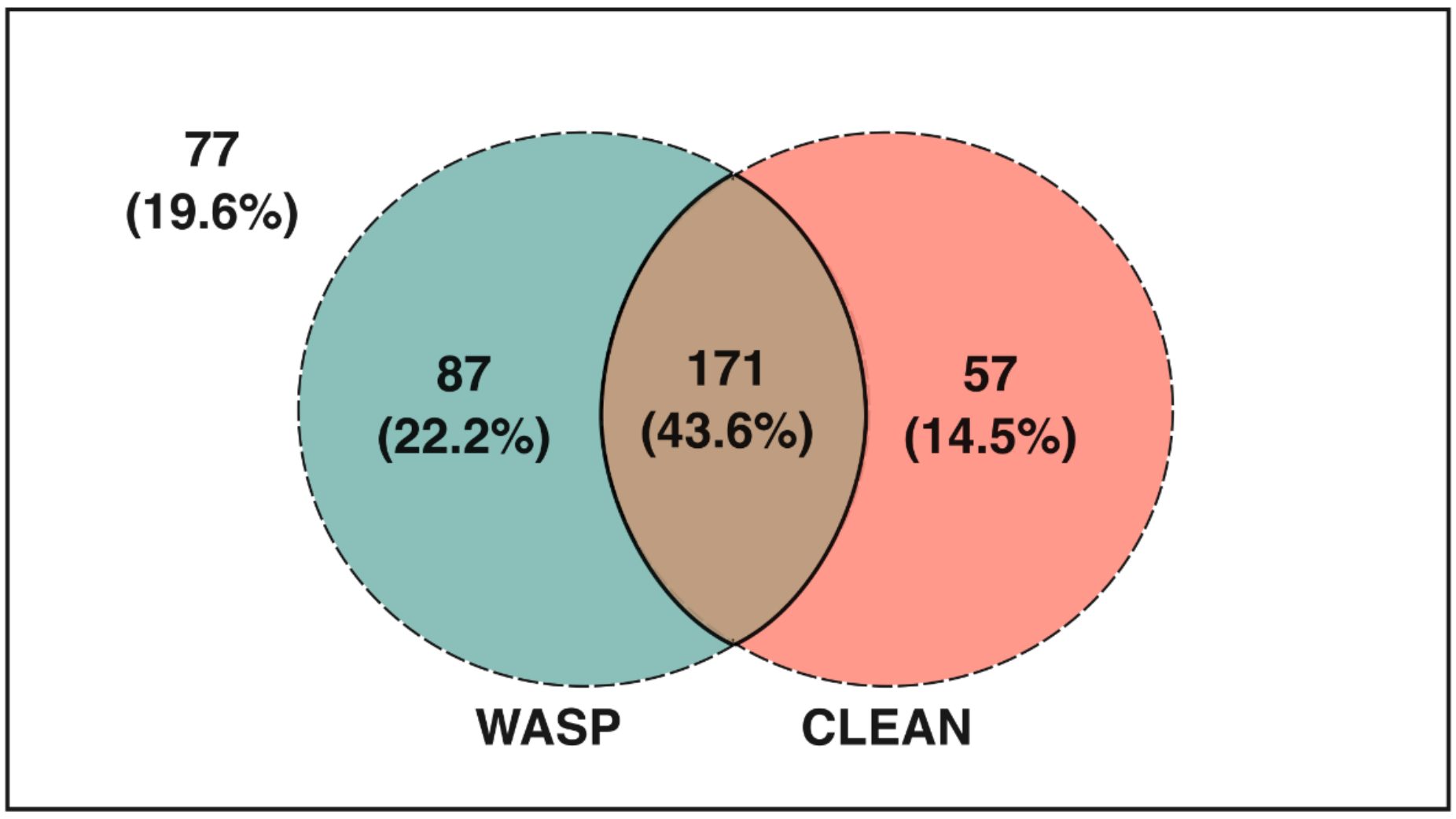

b. Price-149

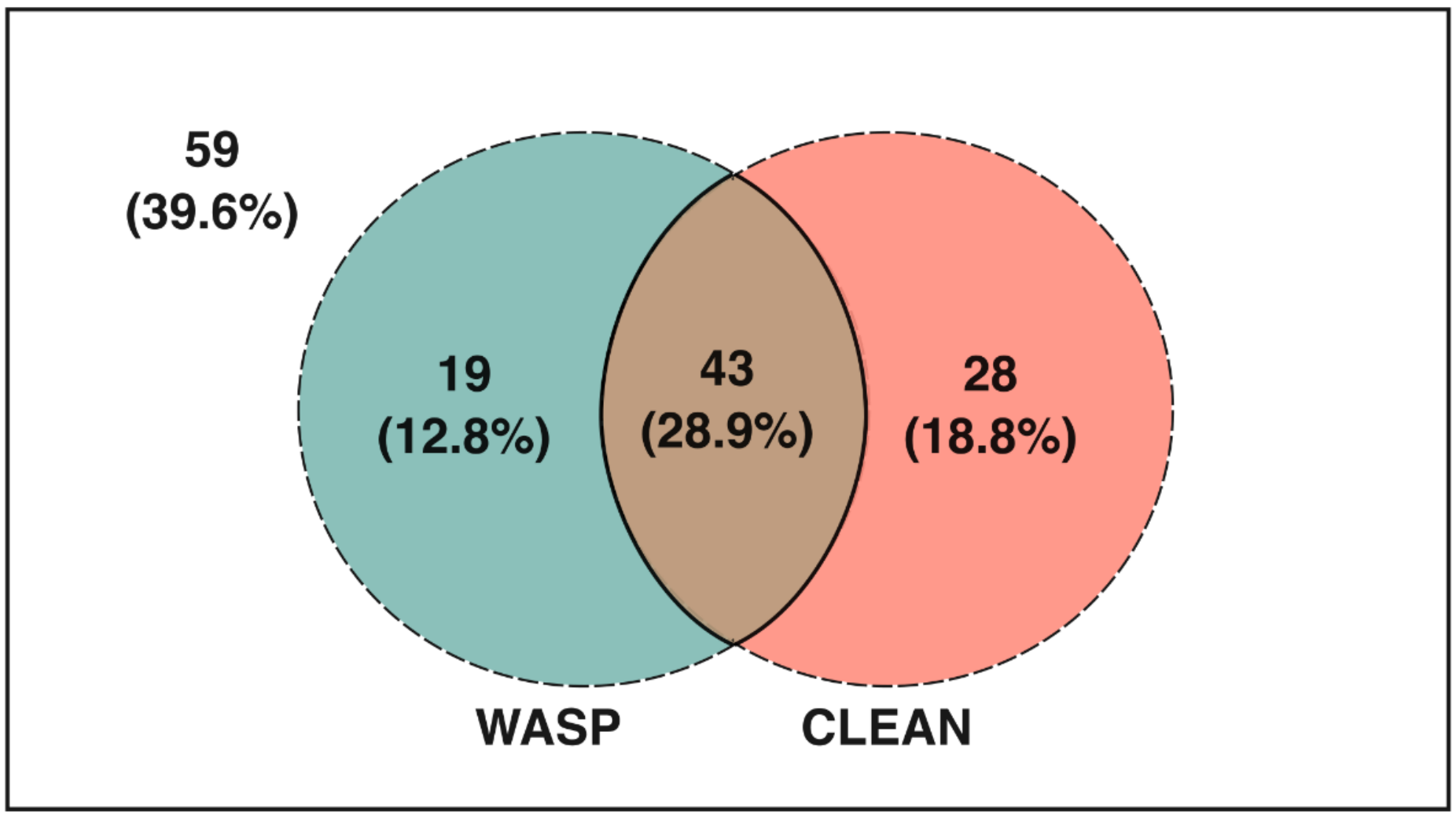

Supplementary Figure 6. Benchmark scores for full four-digit EC numbers (X.X.X.X) for the combined results obtained by WASP and CLEAN, and HiFi-NN on the Price-149 dataset. Scores for HiFi-NN were sourced from ref. (Ayres et al.).

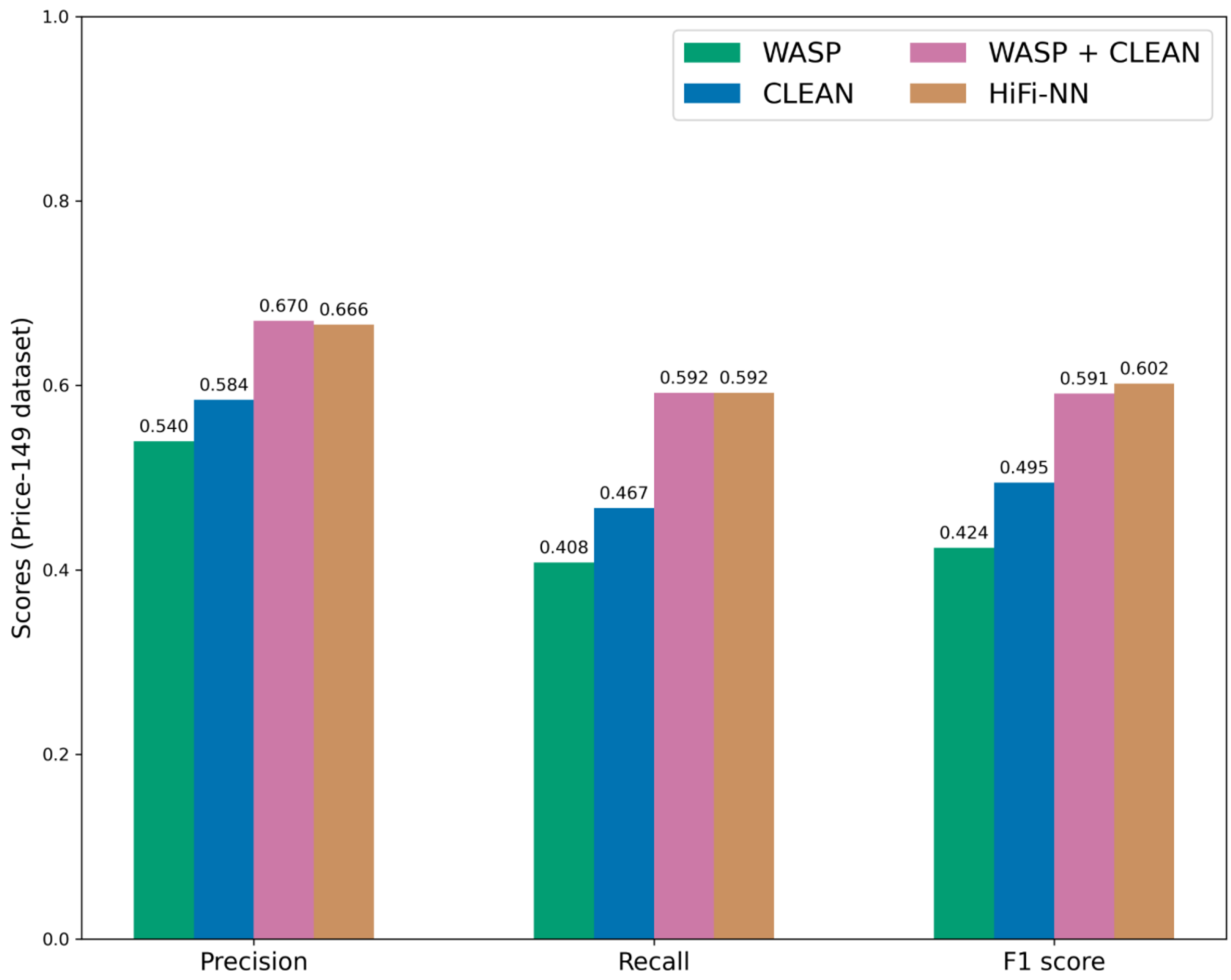

Supplementary Figure 7. Violin plots comparing model quality checks of *Yarrowia lipolytica NRRL Y-64008* predicted by ColabFold, showing distributions of average predicted Template Modelling score (pTM) and average predicted Local Distance Difference Test (pLDDT) against *Yarrowia lipolytica CLIB-122*.

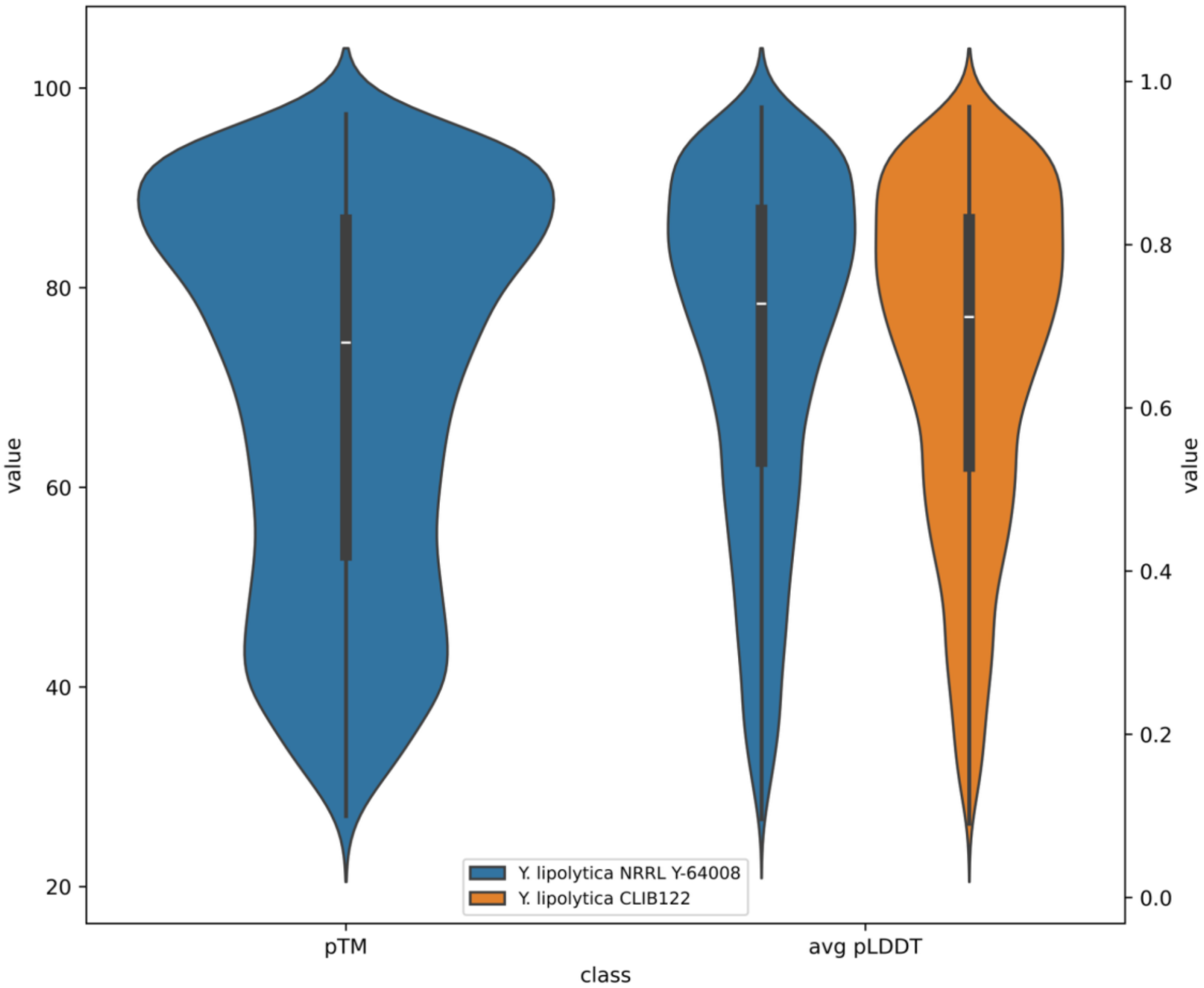

Supplementary Figure 8. Annotation statistics for the *Y. lipolytica* NRRL Y-64008 proteome using WASP. The heatmap shows predicted labels (blue) and no labels (orange) for UniProt IDs across the six functional descriptors. The cumulative count plot above summarises the total annotations per descriptor. The vertical bar plot on the right indicates the total proteome size.

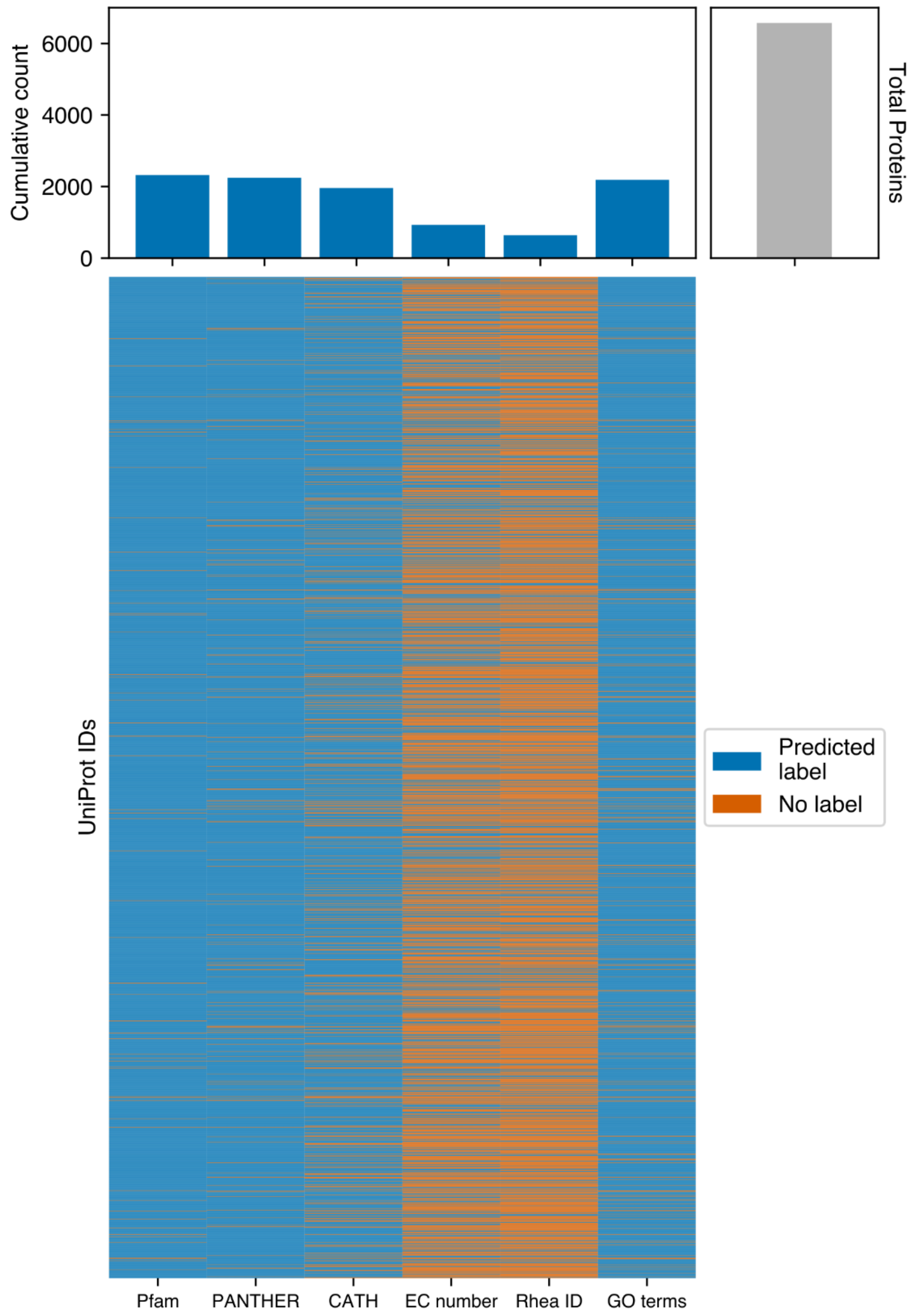

Supplementary Figure 9. Functional map of the *Y. lipolytica* NRRL Y-64008 proteome produced by SAFE, showing clusters with more than 50 nodes. Each cluster is annotated with the five most frequent words from the associated GO terms, illustrating the main functional themes.

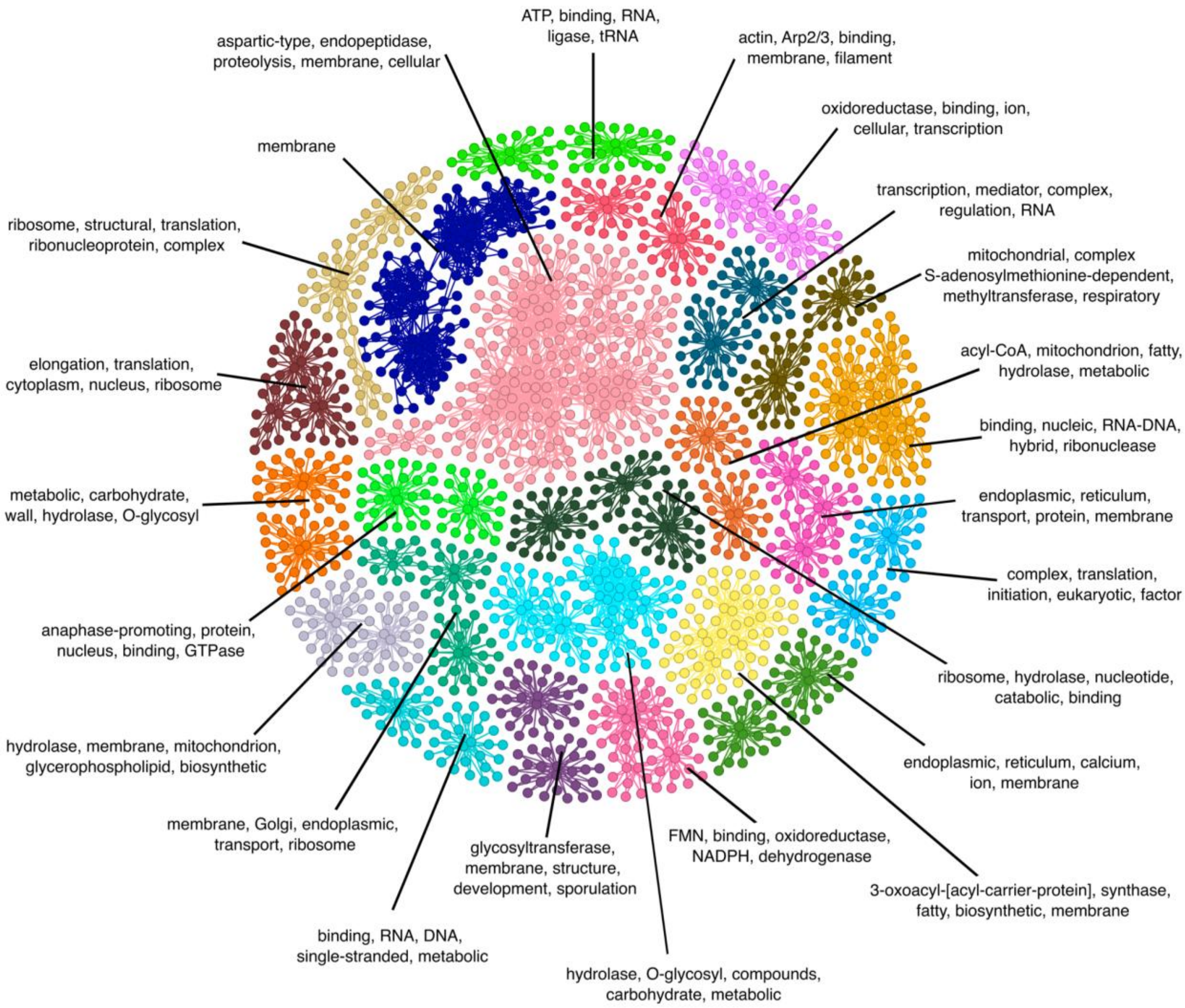

Supplementary Figure 10. Enrichment dot plot showing the distribution of GO terms and EC numbers for the *Y. lipolytica* NRRL Y-64008 proteome. The left panel displays GO terms with bubble size representing the count of proteins associated with a specific GO pathway. The right panel shows EC numbers, with bubble size indicating the count of proteins classified under each enzyme category.

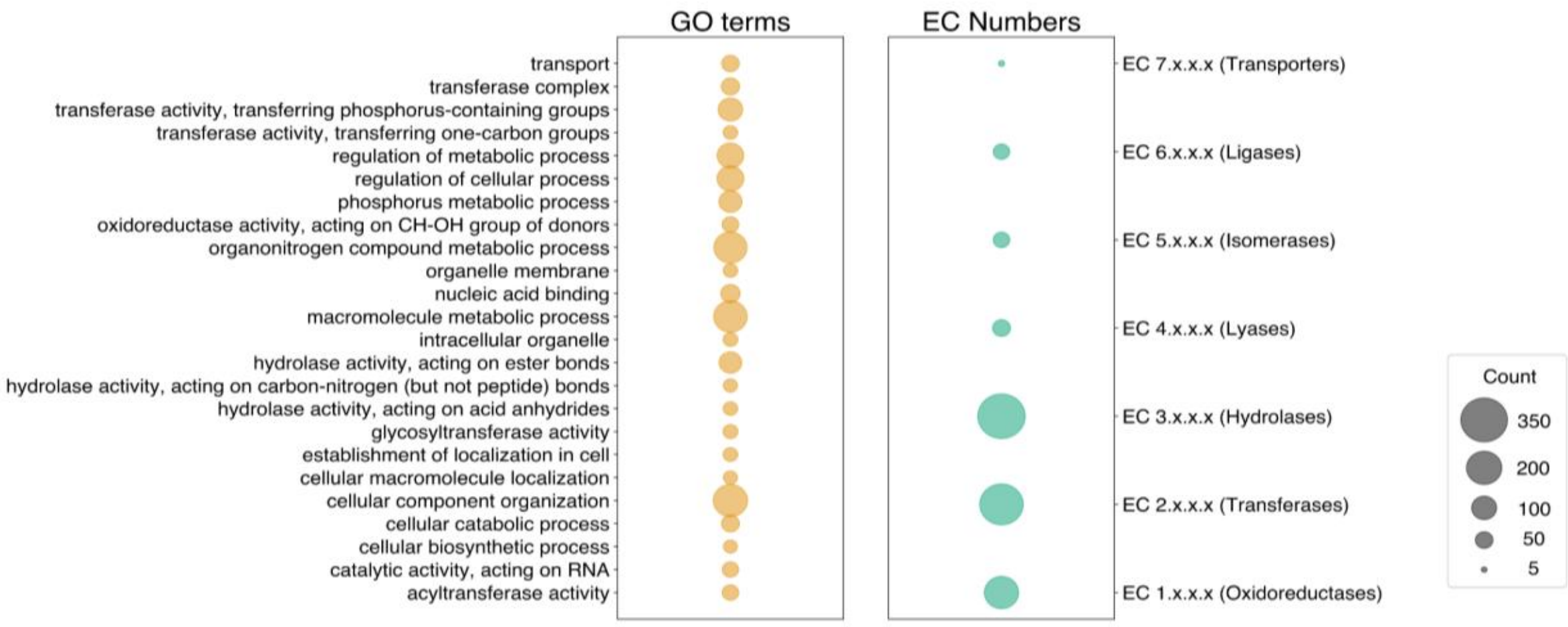

Supplementary Figure 11. Upset plot showing the annotation prediction agreement among WASP, BLAST, and EggNOG predictions for the *Y. lipolytica* NRRL Y-64008 proteome for the GO terms functional descriptor. The bar chart quantifies the number of predictions per agreement category, while the intersection matrix details the specific combinations of method agreements.

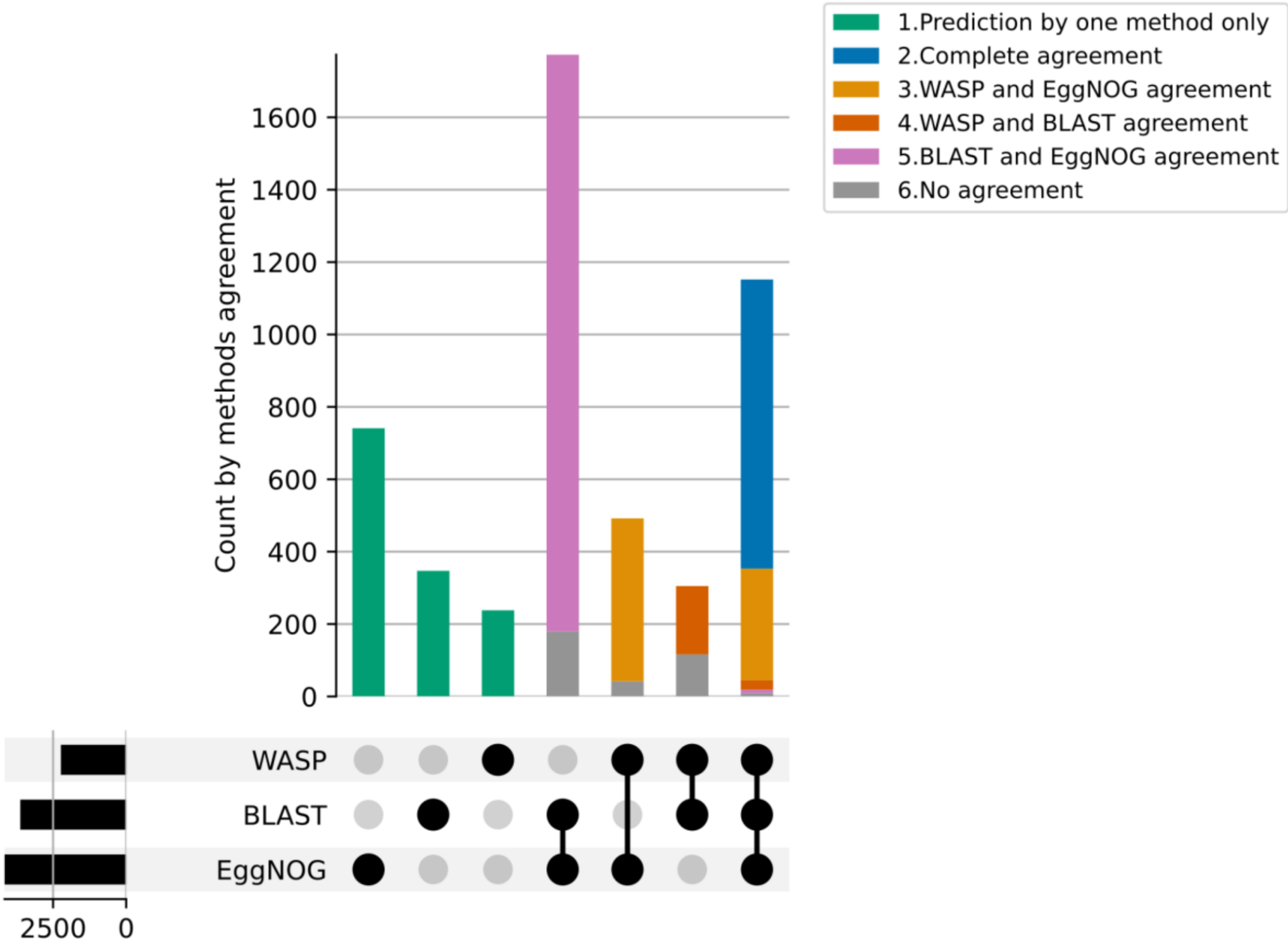

Supplementary Figure 12. Upset plot showing the annotation prediction agreement among WASP, BLAST, and EggNOG (**a**) and CLEAN (**b**) for the *Y. lipolytica* NRRL Y-64008 proteome for the EC number functional descriptor. The bar chart quantifies the number of predictions per agreement category, while the intersection matrix details the specific combinations of method agreements.

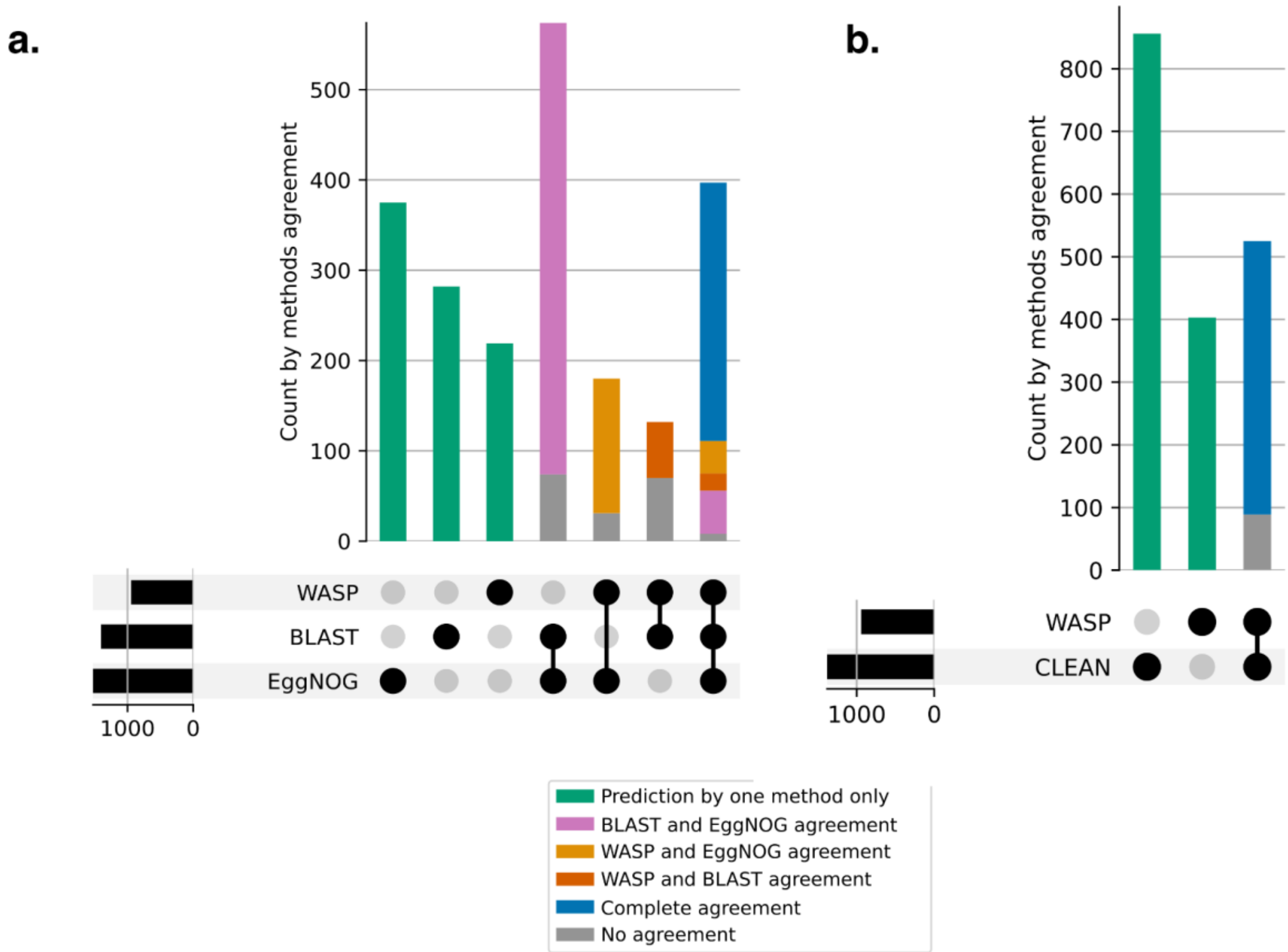

Supplementary Figure 13. Phylogenetic tree displaying the distribution of the biotin gene cluster organisation across Dikarya. Organisms highlighted in pink contain a single-domain DAPA aminotransferase, while those in blue possess bifunctional ATP-dependent dethiobiotin synthetase and 7,8-diaminopelargonic acid (DAPA) aminotransferase enzymes.

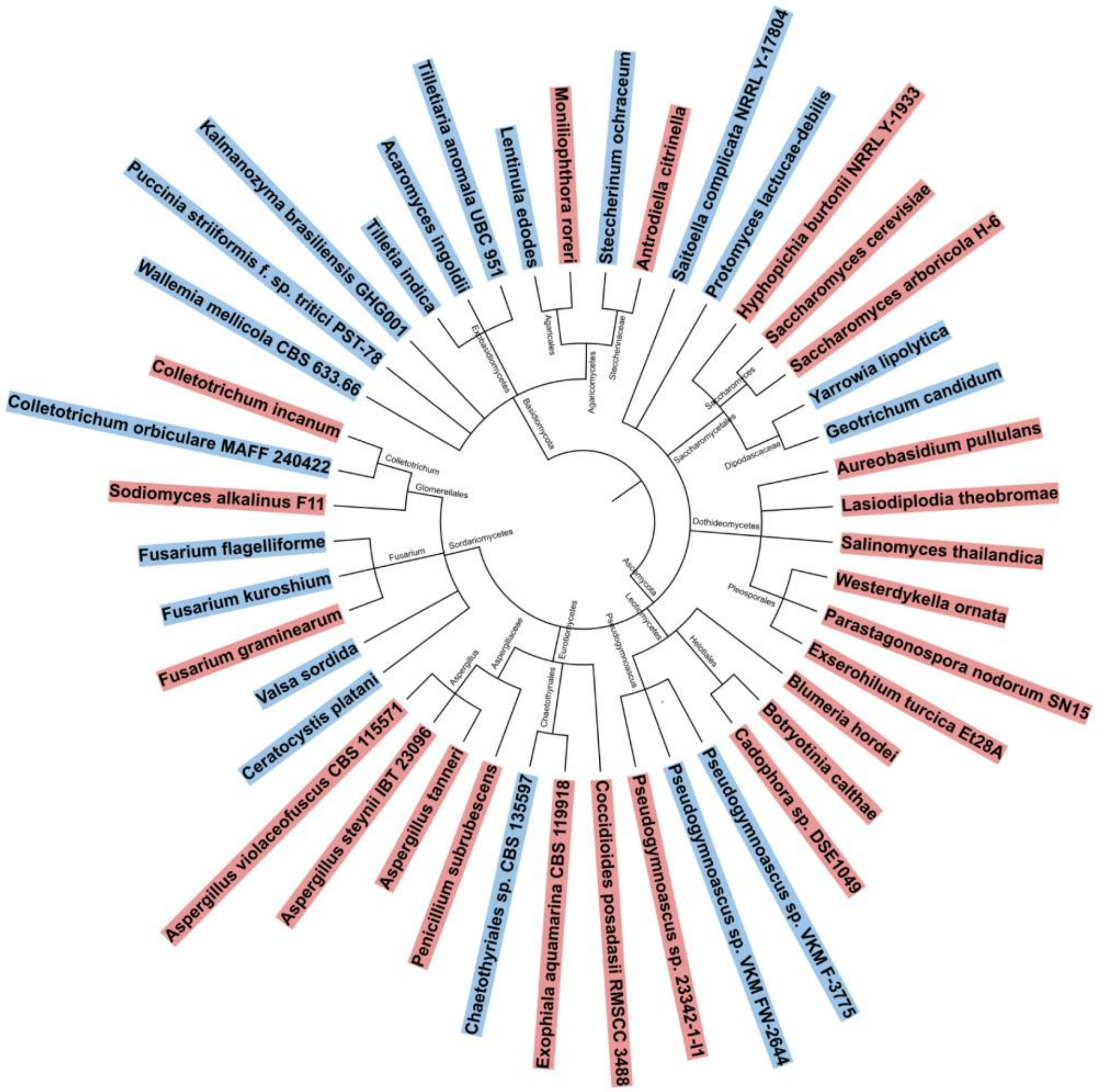

Supplementary Figure 14. Molecular docking of *Mycobacterium tuberculosis* protein Rv2172c with AB131. Docking was performed using AutoDock Vina via the SwissDock platform. Protein backbone is shown in grey ribbon representation, while the ligand AB131 is displayed in ball-and-stick format. Key residues forming the binding pocket are highlighted: positively charged residues (e.g., ARG119, ARG153, ARG159, LYS163, ASP221) are shown in blue, while hydrophobic residues (e.g., MET50, ILE51,VAL88, VAL115, VAL117, PRO118, ILE150) are shown in red. These residues stabilize AB131 through a combination of hydrogen bonding, electrostatic, and hydrophobic interactions. The binding mode suggests that AB131 occupies the NADH pocket of Rv2172c and may inhibit its activity, consistent with the mechanism previously described in *M. smegmatis*.

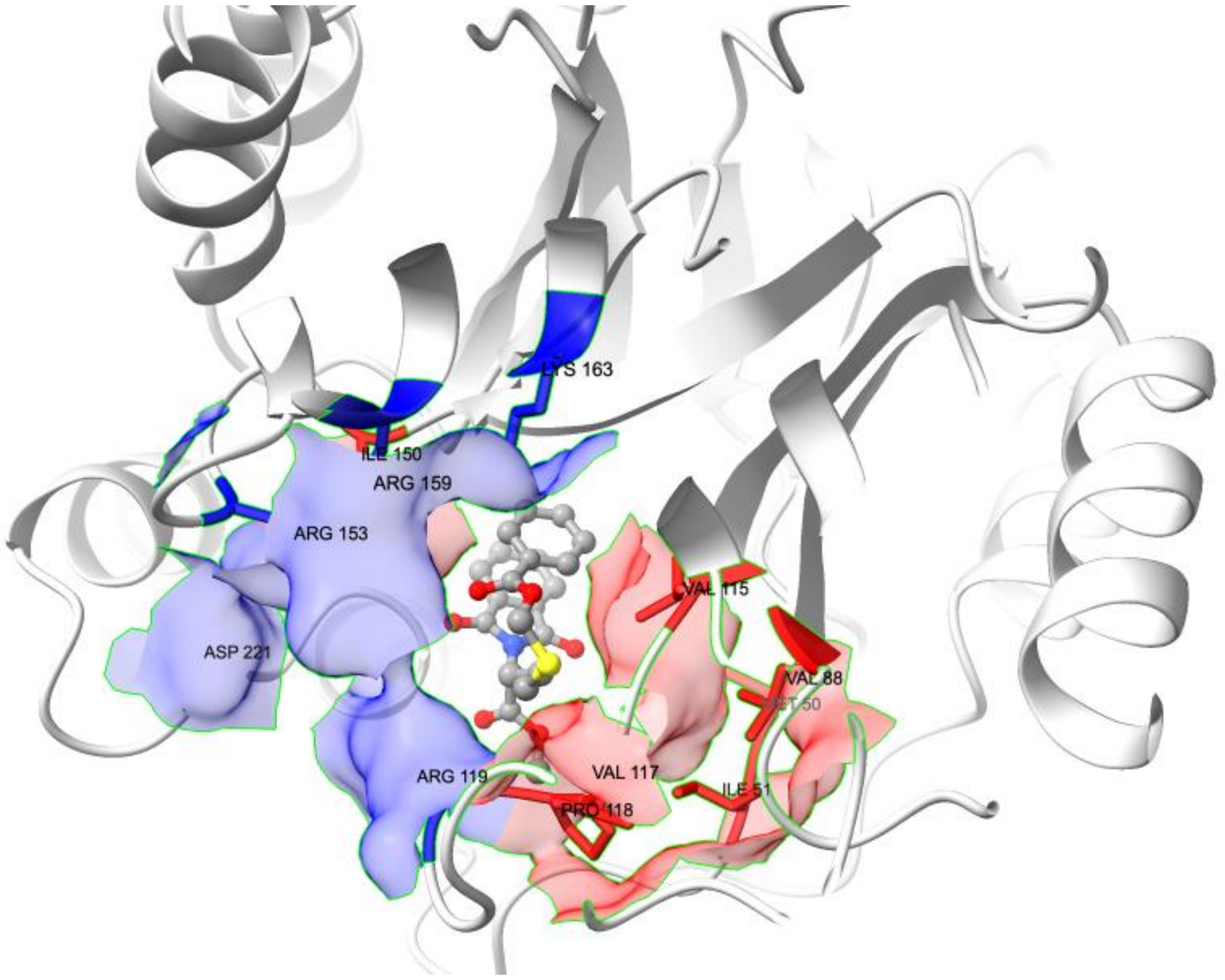

Supplementary Table 1. The table presents the augmented annotations of selected example proteins from *S. cerevisiae* and *E. coli*, highlighting the contributions of WASP in providing new functional insights. Blue cells represent new annotations by WASP, blank cells indicate existing annotations from UniProt, '-' denotes the absence of annotation.

UniProt ID	Pfam	PANTHER	CATH	EC number	Rhea ID	GO terms
P13045	PF10509<br>PF08544<br>PF00288	PTHR10457:<br>SF7	1.20.1440.340<br>3.30.230.10	EC:2.7.1.6	RHEA:13553	GO:0004335<br>GO:0000435<br>GO:0006012
P38616	PF00710	-	3.40.50.40<br>3.40.50.1170	EC:3.5.1.1	-	-
P09932	PF05204<br>PF05203	PTHR43607:<br>SF1	2.170.16.10<br>3.10.28.10	EC:7.1.2.2	RHEA:57720	-
P77297	PF11392	-	3.40.60.261	EC:6.2.1.5	-	GO:0004775<br>GO:0009361<br>GO:0004776<br>GO:0016874
P76148	PF13811	PTHR12544:<br>SF29	3.40.710.10	EC:3.5.1.2	RHEA:15889	GO:0004359<br>GO:0006541

Supplementary Table 2. Distribution of orphan reactions based on the first digit of their EC numbers. The columns distinguish between the number of proteins for which a reference structure in UniProt was identified and those excluded from the analysis due to the lack of a reference structure. The "Unclassified" category includes reactions with unstandardized nomenclature and reactions that couldn't be automatically mapped to an EC category.

EC category	Have a reference protein	Do not have a reference	Total
EC 1.X.X.X	329	432	761
EC 2.X.X.X	591	503	1094
EC 3.X.X.X	299	367	666
EC 4.X.X.X	143	61	204
EC 5.X.X.X	94	29	133
EC 6.X.X.X	284	250	534
EC 7.X.X.X	160	3369	3529
Unclassified	0	520	520
Total	1900	5541	**7441**

Supplementary Note 1.

WASP improves phylogenetic tree reconstruction using phenotypic annotation.

WASP improves the annotation of whole organisms, facilitating a deeper understanding of their phenotypes and metabolic capabilities. We decided to use the improved annotation generated by WASP to conduct a cross-organism comparison using phenotypic data. We expanded our dataset to include a total of 34 organisms from various branches of the tree of life, including plants, animals, protozoans and archaea other than fungi and bacteria previously considered.

We created two binary matrices for each of the six functional descriptors, one based on pre-existing UniProt annotations and the other also incorporating the new annotation obtained from WASP. Each matrix featured annotation labels as columns and organisms as rows, with binary values indicating the presence (1) or absence (0) of at least one protein annotated with the corresponding feature. The matrices were then filtered to remove highly correlated features and features with consistent values across more than 90% of the organisms.

Principal Component Analysis (PCA) on the matrices revealed improved separation of the organisms into the different kingdoms of life when incorporating WASP annotations, particularly for EC numbers and Rhea IDs. We then used hierarchical agglomerative clustering of the PCA results to generate dendrograms, which we compared to a reference phylogenetic tree obtained through NCBI. As a metric to compare the trees we chose the clustering information distance, which measures the amount of information shared between splits of two trees. Dendrograms derived from WASP annotations consistently achieved lower clustering information distances than those based on previous annotation, indicating a closer alignment with the reference phylogenetic tree (**Supplementary Table 3**). The improvement was particularly visible for the EC number and Rhea ID descriptors, highlighting the ability of WASP in normalising protein knowledge across diverse organisms (**Supplementary Figure 15**). These findings demonstrate that WASP significantly enhances the standardisation of functional annotations, reducing discrepancies between well- and poorly-annotated organisms, and thereby leading to a more accurate reconstruction of the tree of life.

	Pfam	PANTHER	CATH	EC number	Rhea ID	GO terms
Before WASP	0.426	0.406	0.370	0.545	0.535	0.564
After WASP	0.398	0.435	0.339	0.370	0.448	0.558

Supplementary Table 3. Clustering information distance against NCBI Taxonomic tree before and after applying WASP.

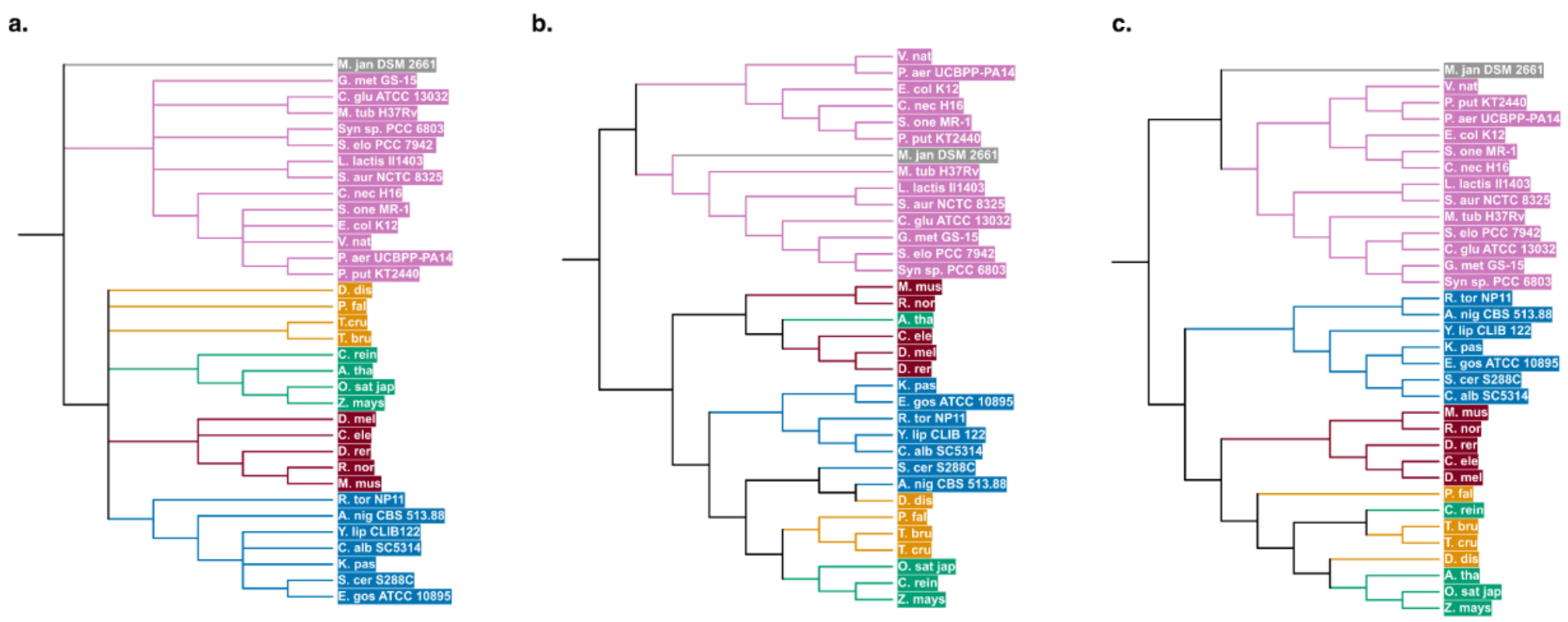

Supplementary Figure 15. NCBI reference phylogenetic tree (**a**), compared to the reconstructed tree before using WASP annotation (**b**) and after (**c**) for the EC number descriptor. (Gray) Archaea, (Pink) Bacteria, (Orange) Protozoan, (Green) Plants, (Red) Animal, (Blue) Fungi.
