# Supplementary material for: WASP: a pipeline for functional annotation prediction based on AlphaFold structural models
Source: Nat Commun. 2026 Jul 28;17:9156. doi: 10.1038/s41467-026-75956-z (PMC13522567; doi:10.1038/s41467-026-75956-z)
Supplement: Supplementary file 2 [file 41467_2026_75956_MOESM2_ESM.pdf]

Description of Additional Supplementary Files

File Name: Supplementary Data 1

Description: Comprehensive list of GTPase FtsZ/Tubulin protein family (CATH Superfamily 3.40.50.1440) proteins from SwissProt used in analysis (.xlsx format).

File Name: Supplementary Data 2

Description: The cohort of 20 model organisms relevant for industrial applications used in this study and links to the corresponding genome-scale metabolic models used for curation (.xlsx format).

File Name: Supplementary Data 3

Description: Complete annotation results statistics for the 20 organisms as determined by WASP (.xlsx format).

File Name: Supplementary Data 4

Description: WASP prediction versus true labels in the Price-149 dataset, where predictions following common reaction rules with the true labels are highlighted (.xlsx format).
