# Supplementary material for: WASP: a pipeline for functional annotation prediction based on AlphaFold structural models
Source: Nat Commun. 2026 Jul 28;17:9156. doi: 10.1038/s41467-026-75956-z (PMC13522567; doi:10.1038/s41467-026-75956-z)
Supplement: Supplementary file 4 [file 41467_2026_75956_MOESM8_ESM.pdf]

nature portfolio

Peer Review File

WASP: A pipeline for functional annotation prediction based on AlphaFold structural models

Corresponding Author: Professor Rodrigo Ledesma-Amaro

This file contains all reviewer reports in order by version, followed by all author rebuttals in order by version.

Version 0:

Reviewer comments:

Reviewer #1

(Remarks to the Author)
Missier et al presents a pipeline for improved annotation of proteins based on structural homology. While the paper is well presented with compelling evidence that WASP can present an improved annotation, I don't see the novelty and critical approach with validation in this study. There is no new tools or method generated as part of the study. Regarding integrating the structure protein to the GEMs, while it is appealing, but not novel. The important step is performing in depth computational analysis, experimental/functional/structural validation or biochemical assays.

My main concern lies within the novelty of the pipeline itself. The tool that the authors present retrieves the structures of a target organism from a public repository, it then employs FoldSeek and network analysis (networkx) and then performs annotation using public databases.

Although the authors are correct in comparing WASP to other similar methods that only uses sequence-based methods, and that these types of tools are needed in the community, the novelty of WASP is unclear to me, other than just a set of tools together. If the major value that the tool presents is a new pipeline, then the authors should improve their efforts to make it more user-friendly for non-bioinformaticians (that could easily write such a pipeline themselves), and also adding more in-depth computational analysis. This could be done either by developing a web-based application or make it easier to implement through a Nextflow pipeline. Alternatively, the authors should make a bigger effort to show the biological value of the tool if it is kept in its current state. The manuscript does not currently involve any experimental validations and could be included to show that some of the predictions are true.

The use of WASP to genome scale metabolic models (GEMs) to improve orphan reactions is an interesting application. Although it is useful to have genes / proteins associated with all metabolic reactions, the authors should then attempt to show how this improves GEM predictions through constraint-based modelling. This could show the biological meaningful application of using WASP. One really need to show how the predictivity and constraining of GEMs improved.

Reviewer #2

(Remarks to the Author)
I co-reviewed this manuscript with one of the reviewers who provided the listed reports. This is part of the Nature Communications initiative to facilitate training in peer review and to provide appropriate recognition for Early Career Researchers who co-review manuscripts.

Reviewer #3

(Remarks to the Author)
Missier et al. present a computational pipeline called WASP for protein functional annotation, which combines AlphaFold with other algorithms to detect homologs based on structural information. The method is benchmarked on several datasets and outperforms sequence-based approaches in recovering various functional descriptors. WASP is applied to 20 model organisms, improving the annotation of 20-30% of previously uncharacterized proteins. Additionally, the method can be

adapted to identify native enzymes responsible for “orphan reactions,” which are reactions without known associated enzymes. The authors conclude that incorporating structural homology is crucial for identifying novel annotations that sequence-based methods may miss.

The authors have conducted a comprehensive evaluation of their method. Overall, the approach and analysis are sound, and the results are compelling. However, there are several issues that prevent the paper from being ready for publication in its current form, which are summarized as follows.

Using AlphaFold to identify remote structural homologs for improving functional annotation is a promising, yet not entirely new, approach. For example, the FASSO method (Andorf et al., bioRxiv, 2022, https://doi.org/10.1101/2022.11.10.516002) employs a similar concept, combining AlphaFold and FoldSeek to detect homologs for genome-wide functional annotations. Other methods following this approach include MorF (Ruperti et al., Genome Biology, 2023, https://doi.org/10.1186/s13059-023-02942-9), PARSE (Derry and Altman, bioRxiv, 2023, https://doi.org/10.1101/2023.10.13.562298), and 3DFI (Julian et al., Bioinformatics Advances, 2021, https://doi.org/10.1093/bioadv/vbab030). Unfortunately, the authors do not reference these previous efforts, making it unclear what the novelty of their work is and how it compares to these existing structure-based methods.

The paper lacks clarity and requires improvement. For example:
- It is unclear how the network is constructed. On page 19, the phrase "a protein is found in the neighborhood…" is ambiguous—does it refer to the query protein or its best hit?
- Why are iterations necessary instead of directly setting n=30?
- What global topology of the graph is used, and how is it applied?
- The inconsistent use of the term "target" throughout the manuscript is also confusing. For example, "target organism" often refers to the query, while "target structures" refers to structures from the databases, not the query. Consistency in terminology would improve clarity.

Reviewer #4

(Remarks to the Author)
In the manuscript by Missier et al. the authors develop WASP, a protein function prediction pipeline. WASP utilizes Foldseek, which searches for proteins in AlphaFold database with similar structure to a query with a PDB resolved 3D structure, to generate a network of shared structural homology. The authors utilize the network to generate structure-based homology clusters and leverage functional annotations. Proteins are then assigned with a function by their neighbors. WASP’s performance is evaluated using the New-392 and experimentally validated Price-149 datasets, and compared to protein sequence-based methods which predict protein function for unannotated proteomes.

While the use of AlphaFold inferred structural homology for function prediction is certainly novel and interesting, it seems that WASP is doing something different in practice, whereas the writing and comparisons are confusing and misleading. WASP in its current form (as far as we could understand) uses the actual 3D structure of proteins from PDB as input, which makes it applicable to only a small subset of proteins with resolved structure, and not to proteins with unknown function. The comparison against methods that uses only the protein sequence as input is therefore invalid.
In a later analysis, the author used predicted protein 3D structure via colabfold as input, instead of the resolved PDB structure, but this pipeline was never evaluated and benchmarked. If the authors can show that a pipeline using only the predicted 3D structure performs similarly well, this tool may become useful, and the comparisons performed could become valid.

Major comments:
1. The input used for WASP is not clearly specified and the description is misleading. Our understanding is that WASP is applied proteins from PDB with the resolved PDB structure, which are used as input for all benchmarking comparisons (e.g. Fig 3). However, the abstract states: “With the emergence of AlphaFold, rapid generation of structural models are offering a new paradigm for inferring protein function based on structural homology” implying that the authors use the predicted structure from sequence, which is misleading.
1. A method that takes as input the experimentally resolved 3D structure is not useful because most proteins with resolved structure have a well annotated function, and only a small subset of the protein have a resolved 3D structure. Therefore, the PDB 3D structure should not be used as input if the authors claim that the method is useful to predict function for proteins of unknown function.
2. In contrast, when the authors predict the function for uncharacterized proteins, they use the predicted 3D structure using colabfold. This is certainly an interesting setup, if it works. However, as far as we could understand, this method was never evaluated in the benchmarking comparisons against CLEAN, ProteInfer, DeepEC and EggNOG-mapper. For this analysis to become valid, the authors should benchmark a method that uses colabfold predicted 3D structure, rather than the PDB 3D structure. This would not only make the method applicable to proteins with unknown functions, but also allow a fair comparison against methods that use only protein sequences as input.
3. The use of the word “annotation” is incorrect. A method that suggests function for proteins with unknown function is predicting the function, not annotating it. WASP is a protein function prediction method, in similar to the methods it compares against (e.g. CLEAN).
4. In Fig 3d of the performances have 2 different bars (with and without a stroke, which are not defined in the text). In the bars corresponding to “EC X.X.X.X”, CLEAN clearly outperforms WASP, which is misleading. Fig S6 also seems to be using the EC X.X.X.X annotations. Therefore, WASP does not actually outperforms on this dataset, although it uses more information.

5. The reference list is missing

Minor:
1. Please check for typos, e.g. Supp Table 2 has “EC 1.X.X.X” appearing twice.
2. The word “model” usage is confusing

Reviewer #5

(Remarks to the Author)
I co-reviewed this manuscript with one of the reviewers who provided the listed reports. This is part of the Nature Communications initiative to facilitate training in peer review and to provide appropriate recognition for Early Career Researchers who co-review manuscripts.

Reviewer #6

(Remarks to the Author)
I co-reviewed this manuscript with one of the reviewers who provided the listed reports. This is part of the Nature Communications initiative to facilitate training in peer review and to provide appropriate recognition for Early Career Researchers who co-review manuscripts.

Version 1:

Reviewer comments:

Reviewer #1

(Remarks to the Author)
The main concern remains - the overall novelty of the pipeline. While the additional analyses have improved the rigor of the workflow, the innovation, novelty, and discovery aspects of the tool are still not evident at a high impact level. Comments on the integration of this on the GEMs and improvement of the GEMs prediction, constraint-based analysis and network-dependent analysis stays without specific response and the new analysis doesn't answer the comment.

(Remarks on code availability)

Reviewer #2

(Remarks to the Author)
I co-reviewed this manuscript with one of the reviewers who provided the listed reports. This is part of the Nature Communications initiative to facilitate training in peer review and to provide appropriate recognition for Early Career Researchers who co-review manuscripts.

(Remarks on code availability)

Reviewer #3

(Remarks to the Author)
The authors have made great efforts to address my previous concerns. I have only one minor point to raise: it would be helpful to define “BH transfer” either in the Figure 3 legend or in the main text (page 9) where it is first introduced.

Otherwise, this is a well-executed study, and the presentation is clear and well written.

(Remarks on code availability)
The repository is well documented. By following the instructions, I can install the package easily.
However, when running the example code, I got the following error message:

```
> wasp-run -t 559292

Setting required variables:

Selected taxid is: 559292
Selected evalue threshold is: 1e-10
Selected bitscore threshold is: 50
Traceback (most recent call last):
File ".../wasp-env/bin/wasp-run", line 8, in <module>
```

```
sys.exit(main())
^^^^^^
File ".../wasp-env/lib/python3.11/site-packages/wasp/main/run.py", line 73, in main
print(f"Selected max neighbours is: {args.max_n}")
^^^^^^^^^^
AttributeError: 'Namespace' object has no attribute 'max_n'. Did you mean: 'max_n '?
```

Reviewer #4

(Remarks to the Author)
The authors have substantially improved the manuscript in revision. There are some remaining issues to improve its utility and clarity.

1) The distinction between functional prediction and annotation should be made clear. The name "functional annotation" without the word prediction in the title and tool name is highly misleading. In the examples referred to by the authors, this distinction is made clear. E.g. in https://link.springer.com/article/10.1186/s13059-019-1835-8 explicitly refers to the term "Term-centric prediction of experimental annotations", and https://www.science.org/doi/10.1126/science.adf2465 uses "Enzyme function prediction" in the title. In these cases, annotation is treated as the benchmarking target or challenge, while the methods themselves generate predictions of annotations.
WASP performs function prediction, or more precisely, prediction of functional annotations, and this should be clearly stated in the tool name, title, and description to accurately convey its scope and to avoid conflating predicted functions with curated or experimentally validated annotations. One cannot simply refer to predictions as annotations, as these are distinct terms with different meanings in the field.
2) As this manuscript includes benchmarking analyses, the authors must provide complete and executable scripts to allow accurate reproduction of all analyses and benchmarking comparisons presented in the paper. Although the authors indicate in the code and Software Submission Checklist that these materials are provided, we could not find the corresponding scripts in the GitHub repository or elsewhere. Making these scripts publicly available is essential for reproducibility and for proper evaluation of the study.
3) A full main figure (Fig. 2) is devoted to a single illustrative example. While this serves as a useful implication of utility, the analysis would be much stronger if the authors generalized this observation across a larger set of protein families. In particular, it would be informative to assess how frequently similar cases occur, and to characterize the features that distinguish such cases from those where simpler approaches suffice
4) More specifically, for the vast majority of cases, sequence homology alone is sufficient for accurate prediction of functional annotations. In such cases, the additional structural prediction step may be unnecessary and computationally wasteful. It would therefore be useful for the authors to provide an analysis offering clearer guidelines on when WASP provides added value over existing homology-based approaches, or to include an evaluation step that determines when sequence homology alone may be insufficient.

(Remarks on code availability)
Most critically, the code required to reproduce the analyses presented in the manuscript is not provided, preventing independent validation of the results.
In addition, in this reviewer's opinion, the practical utility of the method is limited by the complexity of installation, which requires numerous non-trivial dependencies and substantial computational resources.

Reviewer #5

(Remarks to the Author)
I co-reviewed this manuscript with one of the reviewers who provided the listed reports. This is part of the Nature Communications initiative to facilitate training in peer review and to provide appropriate recognition for Early Career Researchers who co-review manuscripts.

(Remarks on code availability)

Reviewer #6

(Remarks to the Author)
I co-reviewed this manuscript with one of the reviewers who provided the listed reports. This is part of the Nature Communications initiative to facilitate training in peer review and to provide appropriate recognition for Early Career Researchers who co-review manuscripts.

(Remarks on code availability)

Version 2:

Reviewer comments:

Reviewer #4

(Remarks to the Author)
Most concerns have been addressed, and the manuscript is substantially improved. One issue that remains insufficiently resolved is the practical utility of the work. In its current form, the contribution is presented primarily as a tool that integrates existing algorithms, but its functionality depends heavily on the availability and versioning of external methods (e.g., Foldseek). This creates a significant limitation, as the long-term usability of the tool may be compromised by changes in these dependencies, while the conceptual advance beyond integration is relatively modest. To ensure robustness and sustained utility in a rapidly evolving field, the authors should provide a fully reproducible deployment framework, such as a Docker container or a complete, version-pinned Conda environment. This would guarantee consistent installation, reproducibility, and continued usability as underlying tools evolve.

(Remarks on code availability)
I was unable to install the tool as provided. The code is not self-contained and instead redirects the user to external dependencies (e.g., Foldseek), without sufficient guidance or integration. This makes installation non-trivial and limits reproducibility.
To address this, the authors should provide a fully self-contained implementation or, at minimum, a clearly documented and version-pinned environment (e.g., Docker or Conda) that includes all required dependencies and ensures straightforward installation and execution.

Version 3:

Reviewer comments:

Reviewer #4

(Remarks to the Author)
The authors addressed my concerns, except that the current solution still leaves the package fully dependent on the continued availability of the authors' GitHub repository. To ensure long-term accessibility and reproducibility, the software should also be deposited in a versioned, immutable archive (e.g., Zenodo or Figshare) matched to the GitHub release.

(Remarks on code availability)
The current solution still leaves the package fully dependent on the continued availability of the authors' GitHub repository. Because GitHub repositories can be altered or removed, I recommend that the editorial team require deposition of a versioned, immutable archive (e.g., Zenodo or Figshare or equivalent) corresponding to the GitHub release to ensure long-term accessibility, reproducibility, and preservation of the software environment.

Open Access This Peer Review File is licensed under a Creative Commons Attribution 4.0 International License, which permits use, sharing, adaptation, distribution and reproduction in any medium or format, as long as you give appropriate credit to the original author(s) and the source, provide a link to the Creative Commons license, and indicate if changes were made.

In cases where reviewers are anonymous, credit should be given to 'Anonymous Referee' and the source.
The images or other third party material in this Peer Review File are included in the article's Creative Commons license, unless indicated otherwise in a credit line to the material. If material is not included in the article's Creative Commons license and your intended use is not permitted by statutory regulation or exceeds the permitted use, you will need to obtain permission directly from the copyright holder.
To view a copy of this license, visit https://creativecommons.org/licenses/by/4.0/

We would like to thank the reviewers for time dedicated to read our manuscript and for the valuable feedback provided. We have addressed all the comments, which can be found in the point-by-point response in this document. As a general summary, we have made the following major improvements:

- Benchmarking and methodological clarity
  WASP now clearly states reliance only on AlphaFold/ColabFold structures. The revised figures and text now show that WASP consistently achieves high precision and recall, particularly on under-annotated datasets, and complements sequence-based methods at finer EC resolution. A comparison with recent methods (FASSO, MorF, 3DFI, PARSE) highlights WASP's unique network-based approach and resistance to misannotation propagation.
- Biological utility
  WASP predictions were integrated into genome-scale metabolic models for three pathogens, resulting in improved gene essentiality prediction accuracy across all species (e.g. in M. tuberculosis, precision increased from 0.82 to 0.92). WASP also corrected false essentiality calls by updating GPR rules.
- Novel insights
  WASP identified new candidate genes linked to biologically relevant and druggable pathways (e.g. Rv1202, Rv2141c, Rv2172c), providing testable functional hypotheses.
- Improved accessibility
  WASP is now available as a pip-installable Python package with full documentation, examples, and troubleshooting guidance.

Reviewer #1 (Remarks to the Author)

Del Missier et al presents a pipeline for improved annotation of proteins based on structural homology. While the paper is well presented with compelling evidence that WASP can present an improved annotation, I don't see the novelty and critical approach with validation in this study. There is no new tools or method generated as part of the study. Regarding integrating the structure protein to the GEMs, while it is appealing, but not novel. The important step is performing in depth computational analysis, experimental/functional/structural validation or biochemical assays.

My main concern lies within the novelty of the pipeline itself. The tool that the authors present retrieves the structures of a target organism from a public repository, it then employs FoldSeek and network analysis (networkx) and then performs annotation using public databases.

Although the authors are correct in comparing WASP to other similar methods that only use sequence-based methods, and that these types of tools are needed in the community, the novelty of WASP is unclear to me, other than just a set of tools together. If the major value that the tool presents is a new pipeline, then the authors should improve their efforts to make it more user-friendly for non-bioinformaticians (that could easily write such a pipeline themselves), and also adding more in-depth computational analysis. This could be done either by developing a web-based application or make it easier to implement through a Nextflow pipeline.

We thank the reviewer for this insightful comment and for the opportunity to clarify the novelty of our work. We agree that our pipeline leverages several well-established and robust tools. However, we respectfully argue that the novelty of a computational pipeline often arises from the thoughtful integration of these components to create a workflow that addresses a new or previously unmet challenge.

The primary innovation of our pipeline is that it provides the first end-to-end, automated solution for achieving protein annotations based on structural insight. Prior to our work, this type of analysis was fragmented and required significant manual intervention, making it inaccessible to many researchers. By fulfilling this clear and unmet need, our work provides a novel, timely, and valuable contribution to the field.

In the reviewed manuscript, we now also demonstrate how WASP-derived annotations improve genome-scale metabolic reconstructions and uncover biologically meaningful and druggable targets, which further highlighting its practical utility and translational relevance (pages 17-18, under new section "WASP reconciles *in silico* essentiality prediction with loss-of-function screens", methods at page 25 and new Supplementary Figure 14).

To address the reviewer's concerns, we have now made the following improvements to our manuscript :
- The tool is now distributed as a standard Python package installable via pip (`pip install wasp-proteins-annotation`), making deployment more easily accessible across different computing environments. The only required input is an NCBI organism ID, with comprehensive documentation provided for more advanced use cases involving custom protein sets. We consciously designed WASP to maintain flexibility in its structure prediction component. While the pipeline optionally integrates ColabFold for structure prediction, we opted to keep the ColabFold installation separate to accommodate the wide variability in users' computational environments (particularly regarding GPU availability and CUDA configurations). This approach follows the precedent set by other widely-used structural bioinformatics tools. Detailed, platform-specific installation guides are provided on ColabFold GitHub to help users set up this component.
- The GitHub repository (https://github.com/gioodm/wasp-proteins-annotation) includes extensive documentation with example files and troubleshooting guides, and we are committed to maintaining active support through the issue tracker.

These improvements make WASP accessible to both bioinformaticians and researchers with limited computational expertise while maintaining the flexibility needed for advanced use cases. The pipeline integrates multiple optimized steps - from homology detection to structure-function mapping - that collectively improve prediction accuracy and robustness. While experienced bioinformaticians could theoretically implement similar workflows, WASP provides pre-optimized, rigorously tested scripts that would otherwise require substantial time and effort to develop and validate individually.

Alternatively, the authors should make a bigger effort to show the biological value of the tool if it is kept in its current state. The manuscript does not currently involve any experimental validations and could be included to show that some of the predictions are true.

The use of WASP to genome scale metabolic models (GEMs) to improve orphan reactions is an interesting application. Although it is useful to have genes / proteins associated with all metabolic reactions, the authors should then attempt to show how this improves GEM predictions through constraint-based modelling. This could show the biological meaningful application of using WASP. One really need to show how the predictivity and constraining of GEMs improved.

We thank the reviewer and, following their comments, we have gathered additional examples showcasing the utility and insight of WASP. We performed additional analyses integrating WASP-predicted protein annotations into genome-scale metabolic models (GEMs) of three pathogenic organisms—*Helicobacter pylori*, *Pseudomonas aeruginosa*, and *Mycobacterium tuberculosis*—all supported by high-quality transposon mutant datasets. We benchmarked gene essentiality predictions before and after WASP refinement against experimental data, assessing improvements in metrics such as precision and F1 score. WASP was also used to resolve false positive essentiality predictions by identifying unannotated homologs and updating gene–protein–reaction (GPR) rules accordingly. These refinements improved GEM model accuracy across all three organisms, with particularly notable gains in *M. tuberculosis*, where precision increased from 0.82 to 0.92 and the F1 score from 0.77 to 0.81.

Importantly, WASP provides a high-throughput and streamlined approach to uncover functional assignments and potential drug targets in silico, building on prior biological knowledge. In fact, after having obtained these refined predictions in *M. tuberculosis*, we explored potential drug targets. The process involved two steps: first, the WASP pipeline automatically suggested new functional annotations for proteins linked to misannotated GPRs and orphan reactions; second, we manually curated these high-throughput results to prioritize candidates that were predicted to be essential in transposon libraries and were listed as high-potential drug targets in databases like UniProt. This systematic approach allowed us to efficiently generate translationally relevant hypotheses with minimal initial experimental effort. For example, Rv1202 and Rv2141c had been suspected to contribute to the acetylornithine deacetylase (ArgE) reaction, and Rv2172c had been implicated in 5,10-methylenetetrahydrofolate reductase (MTHFR) activity, with both reactions considered druggable. While traditional experimental validation of these candidates—through biochemical characterization, mutagenesis, and inhibitor testing—would require substantial time and resources, WASP rapidly and systematically highlights them computationally, generating biologically meaningful and translationally relevant hypotheses in an automated way.These analyses demonstrate that integrating WASP predictions not only enhances GEM accuracy but also enables fast, high-throughput identification of biologically meaningful and druggable enzymes, providing translationally relevant hypotheses with minimal experimental effort.

Full details of these results are presented in the new section “WASP reconciles in silico essentiality prediction with loss-of-function screens” on pages 17–18 of the revised manuscript, the corresponding methods are described on page 25, and a new Supplementary Figure 14 has been included to illustrate the structural and functional insights for one of the top candidates, Rv2172c.

Reviewer #4 (Remarks to the Author)

In the manuscript by Missier et al. the authors develop WASP, a protein function prediction pipeline.

WASP utilizes Foldseek, which searches for proteins in AlphaFold database with similar structure to a query with a PDB resolved 3D structure, to generate a network of shared structural homology. The authors utilize the network to generate structure-based homology clusters and leverage functional annotations. Proteins are then assigned with a function by their neighbours. WASP's performance is evaluated using the New-392 and experimentally validated Price-149 datasets and compared to protein sequence-based methods which predict protein function for unannotated proteomes.

While the use of AlphaFold inferred structural homology for function prediction is certainly novel and interesting, it seems that WASP is doing something different in practice, whereas the writing and comparisons are confusing and misleading. WASP in its current form (as far as we could understand) uses the actual 3D structure of proteins from PDB as input, which makes it applicable to only a small subset of proteins with resolved structure, and not to proteins with unknown function. The comparison against methods that use only the protein sequence as input is therefore invalid. In a later analysis, the author used predicted protein 3D structure via colabfold as input, instead of the resolved PDB structure, but this pipeline was never evaluated and benchmarked. If the authors can show that a pipeline using only the predicted 3D structure performs similarly well, this tool may become useful, and the comparisons performed could become valid.

We thank the reviewer for their assessment of our manuscript and we apologise for the confusion. To clarify the type of inputs used by WASP, all structural models are drawn exclusively from AlphaFold (AFDB) or, when unavailable there, from ColabFold predictions. No PDB-resolved structures are ever used in our pipeline. This is stated in the Methods (Data retrieval, pp. 19–20) and reiterated in the Results (pp. 3–7), where we specify downloading all input structures from AlphaFold DB Google Cloud Bucket for the query organisms and using a pre-clustered, indexed version of AFDB and the curated SwissProt database, obtained through the structural alignment tool Foldseek as database.
To further enhance accessibility and ensure reproducible results, we have also now packaged WASP as a standardised Python library (`wasp-proteins-annotation`), installable via `pip`. This simplifies deployment and execution for end-users.

In the following responses, we expand on this point and detail the additional analyses and improvements we have made to the manuscript to address the reviewer's concerns regarding the pipeline.

Major comments:
1. The input used for WASP is not clearly specified and the description is misleading. Our understanding is that WASP is applied proteins from PDB with the resolved PDB structure, which are used as input for all benchmarking comparisons (e.g. Fig 3). However, the abstract states: "With the emergence of AlphaFold, rapid generation of structural models are offering a new paradigm for inferring protein function based on structural homology" implying that the authors use the predicted structure from sequence, which is misleading.

We apologize for the confusion. We've re-clarified in the text that WASP exclusively uses AlphaFold-predicted structures as input. In the first paragraph of the Results, we state: *"First, WASP fetches protein structure models of the query organism from AlphaFold DB (AFDB) hosted on the Google*

Cloud Bucket."

To avoid any further confusion, in the *Methods* under *WASP implementation details*, we now note: *"AlphaFold structural models for the query organism defined on input through its corresponding NCBI taxonomy ID are downloaded from the AlphaFold Database Google Cloud Bucket through the* `gsutil` *tool. Even when experimentally resolved structures are available in the PDB, WASP uses AlphaFold-predicted models to ensure consistency across the entire proteome."*

1. A method that takes as input the experimentally resolved 3D structure is not useful because most proteins with resolved structure have a well annotated function, and only a small subset of the protein have a resolved 3D structure. Therefore, the PDB 3D structure should not be used as input if the authors claim that the method is useful to predict function for proteins of unknown function.

We agree with the reviewer and as stated above, PDB structures are not an input of WASP.

2. In contrast, when the authors predict the function for uncharacterized proteins, they use the predicted 3D structure using colabfold. This is certainly an interesting setup, if it works. However, as far as we could understand, this method was never evaluated in the benchmarking comparisons against CLEAN, ProteInfer, DeepEC and EggNOG-mapper. For this analysis to become valid, the authors should benchmark a method that uses colabfold predicted 3D structure, rather than the PDB 3D structure. This would not only make the method applicable to proteins with unknown functions, but also allow a fair comparison against methods that use only protein sequences as input.

As mentioned above, all structural models used in WASP—across both benchmarking and novel annotation tasks—are derived from AlphaFold DB or, when necessary, predicted using ColabFold. Therefore, we would like to clarify that the benchmarking presented in the section 'WASP retrieves hidden labels at high accuracy' (Fig. 3) was indeed performed using the setup the reviewer is suggesting. All structural models for the query proteins in the New-392 and Price-149 benchmark datasets were sourced directly from the AlphaFold Database (AFDB). ColabFold, which we use for novel proteomes, is simply the quickest, locally executable version of the AlphaFold2 algorithm and produces structural predictions of equivalent quality to those in AFDB. Therefore, the high performance demonstrated in our benchmarks is directly representative of WASP's capability when applied to proteins with predicted structures, making it fairly comparable to sequence-based methods and fully applicable to proteins of unknown function.

As a consequence, WASP has been rigorously checked against other state-of-the-art tools, and the results validate that a pipeline based on predicted structures is highly competitive for function prediction.

3. The use of the word "annotation" is incorrect. A method that suggests function for proteins with unknown function is predicting the function, not annotating it. WASP is a protein function prediction method, in similar to the methods it compares against (e.g. CLEAN).

We agree that WASP and the methods it compares against perform computational, automated function prediction. In the revised manuscript, we now explicitly acknowledge this distinction when the term is first introduced. Specifically, the Introduction now reads: *"Here we present Whole-*

proteome Annotation through Structural-homology Pipeline (WASP), a python-based pipeline designed for comprehensive organism functional prediction (from here on, referred to as annotation) at the whole-proteome level based on structural homology (Fig. 1a).”
Our use of the term "annotation" to describe computational predictions aligns with standard practice in the field, where it is commonly used in high-throughput functional prediction studies and by major databases and resources (see Zhou et al. Genome Biol 20, 244 (2019), https://doi.org/10.1186/s13059-019-1835-8 , Cantalapiedra et al. Mol Biol Evol 38, 5825–5829 (2021), https://doi.org/10.1093/molbev/msab293 or Yu et al. Science 379, 1358–1363 (2023), https://doi.org/10.1126/science.adf2465). Following the reviewer's comment, we have reviewed the manuscript to clarify this distinction when ambiguous and we are happy to further adjust wording in specific sections if there are passages where the distinction remains unclear.

4. In Fig 3d of the performances have 2 different bars (with and without a stroke, which are not defined in the text). In the bars corresponding to “EC X.X.X.X”, CLEAN clearly outperforms WASP, which is misleading. Fig S6 also seems to be using the EC X.X.X.X annotations. Therefore, WASP does not actually outperform on this dataset, although it uses more information.

Thanks for noticing this. The bar styles in Fig. 3d have now been modified to be defined unambiguously in both the main text and the figure legend. These additions clarify which bar style corresponds to third-digit (X.X.X.-) versus fourth-digit (X.X.X.X) EC performance, and support the claims in the text that WASP excels on the third-digit task while remaining complementary to CLEAN at the fourth-digit level. The legend of Fig. S6 likewise now specifies that it reports four-digit EC (X.X.X.X) predictions.
We now also modified the text to describe more clearly that the manuscript does not claim that WASP outperforms CLEAN for full four-digit EC annotation; rather, we demonstrate that the two methods provide complementary coverage at that more specific level. However, we do highlight that WASP achieves superior performance for third-digit EC number prediction. We further discuss this finding in the context of the EC classification system, which while the field’s standard, can be rigid and may not fully capture enzyme promiscuity or multifunctionality (see page 19). This underlines the value of complementary, structure-based predictions alongside traditional EC assignments.

5. The reference list is missing

We apologize for the error when compiling references and have now added the list at the end of the document.

Minor:
1. Please check for typos, e.g. Supp Table 2 has “EC 1.X.X.X” appearing twice.

We've corrected the duplication of “EC 1.X.X.X” in Supplementary Table 2 and double-checked for any additional typos.

2. The word “model” usage is confusing

We use the term "model" in different contexts throughout the manuscript—to refer to protein structure models (e.g. from AlphaFold), genome-scale metabolic models, and model organisms. We have now revised the text to provide greater clarity in instances where the context was not immediately obvious from the surrounding text.

Reviewer #3 (Remarks to the Author)

Missier et al. present a computational pipeline called WASP for protein functional annotation, which combines AlphaFold with other algorithms to detect homologs based on structural information. The method is benchmarked on several datasets and outperforms sequence-based approaches in recovering various functional descriptors. WASP is applied to 20 model organisms, improving the annotation of 20-30% of previously uncharacterized proteins. Additionally, the method can be adapted to identify native enzymes responsible for "orphan reactions," which are reactions without known associated enzymes. The authors conclude that incorporating structural homology is crucial for identifying novel annotations that sequence-based methods may miss.

The authors have conducted a comprehensive evaluation of their method. Overall, the approach and analysis are sound, and the results are compelling. However, there are several issues that prevent the paper from being ready for publication in its current form, which are summarized as follows.

Using AlphaFold to identify remote structural homologs for improving functional annotation is a promising, yet not entirely new, approach. For example, the FASSO method (Andorf et al., bioRxiv, 2022, https://doi.org/10.1101/2022.11.10.516002) employs a similar concept, combining AlphaFold and FoldSeek to detect homologs for genome-wide functional annotations. Other methods following this approach include MorF (Ruperti et al., Genome Biology, 2023, https://doi.org/10.1186/s13059-023-02942-9), PARSE (Derry and Altman, bioRxiv, 2023, https://doi.org/10.1101/2023.10.13.562298), and 3DFI (Julian et al., Bioinformatics Advances, 2021, https://doi.org/10.1093/bioadv/vbab030). Unfortunately, the authors do not reference these previous efforts, making it unclear what the novelty of their work is and how it compares to these existing structure-based methods.

We thank the reviewer for their assessment of our manuscript and for the additional references, which were valuable. As a result, we now provide a comparative overview of recent AlphaFold-based annotation tools has been added to the Results (page 10). In brief:

- FASSO, MorF and 3DFI employ one-to-one annotation transfers (e.g., from proteome A to proteome B or PDB) without leveraging network topology or enrichment statistics. To benchmark this "standard" approach, we introduced a best-hit baseline (Fig. 3c,d), in which each query inherits all labels from its highest-scoring hit. While this simple transfer excels on the densely annotated New-392 set, it collapses on the sparsely labelled Price-149 dataset.
- WASP, by contrast, performs a proteome-wide search against a clustered AFDB + SwissProt database, applies Reciprocal Best Hit filtering, builds a global homology network, and runs SAFE enrichment over six descriptor types with iterative neighbourhood expansion. This strategy yields both high precision on well-annotated proteins and robust coverage of under-characterized proteins—particularly evident on Price-149.

- PARSE is now cited in the Discussion. It uses pre-trained embeddings of local structural environments combined with statistical enrichment to identify functions with residue-level explainability. Direct benchmarking of PARSE against WASP was not feasible due to software compatibility issues and unavailable support from the authors through GitHub; we nevertheless outline its methodology in the Discussion to acknowledge its relevance.

Significantly, WASP's unique contributions lie in (i) its use of a network-based structural homology framework, (ii) the integration of enrichment statistics for multi-label prediction, and (iii) its demonstrated robustness across both well-annotated and poorly annotated proteomes. These elements, in combination, represent a novel and practical approach to large-scale protein function prediction.

The paper lacks clarity and requires improvement. For example:

Thanks for the comment, we have now revised the whole manuscript and improve clarity when possible. We reply to your specific examples below.

- It is unclear how the network is constructed. On page 19, the phrase "a protein is found in the neighborhood..." is ambiguous—does it refer to the query protein or its best hit?

To improve clarity, we revised the corresponding paragraph in the Methods section (page 19) by explicitly referencing the Reciprocal Best Hit (RBH) strategy and clearly defining "neighbourhood" in both search directions. In this context, "neighbourhood" refers to the set of top hits (by default, the first 10 proteins with bitscore > 50 and e-value < 1e-10) obtained for each protein during structural alignment. A protein pair is retained only if it is a significant match and appears within the defined neighbourhood (i.e., among the top 10 hits by default) of both the original query protein (when searched against the database) and its best hit (when the best hit is used as a reciprocal query and searched against the same database).

- Why are iterations necessary instead of directly setting n=30?

This was to give more weight to early iterations which have a better precision. We have now clarified this point on p.7-8 of the main next (see below) and in the Methods (p. 20). This now reads: "*Iteratively increasing the number of neighbours helps recover annotations for proteins that are initially unlinked or part of low-connectivity regions by introducing new, potentially informative associations. This strategy was introduced to find a balance between precision and recall, ensuring that confident annotations are made early, while enabling broader coverage in later steps for proteins still missing a functional prediction. … Notably, we retained the iterative approach despite the low false positive increase, as it maintains high specificity by prioritising confident, local enrichments before relaxing stringency. All parameters, including the number of iterations and neighbours per iteration, remain customisable by the user to adapt to different annotation goals or dataset characteristics*."

- What global topology of the graph is used, and how is it applied?

In WASP, “global topology” refers to the overall structure of the protein-protein homology network constructed from reciprocal best hit (RBH) pairs, in which nodes represent proteins (from both the query organism and reference databases) and edges are weighted by Foldseek alignment scores (explained at page 5 and in Methods, pages 19-20). This network is used as the basis for annotation via the SAFE algorithm. SAFE applies topological analysis by defining local neighbourhoods for each node — sets of nearby proteins determined by a graph distance threshold — and computing neighbourhood enrichment scores for functional descriptors (GO terms, EC numbers, etc.) using a hypergeometric test. These enrichments are propagated across the graph to assign putative annotations to previously unannotated proteins, as detailed in the original publications (Baryshnikova 2016, 10.1016/j.cels.2016.04.014 and Baryshnikova 2018, 10.1007/978-1-4939-8618-7_12).
Thus, WASP leverages global topology in two ways: (1) it identifies functionally coherent clusters where annotations can be reliably inferred based on connectivity, and (2) it allows information propagation beyond direct hits, enabling annotation of proteins based on their extended local environment in the graph. We now highlight this in the Discussion (page 18).
If the reviewer is instead referring to the graph layout (e.g. force-directed positioning in figures), our algorithm does not depend on any particular layout; visualizations in Figures 1b and 2c are merely for illustration.

- The inconsistent use of the term "target" throughout the manuscript is also confusing. For example, "target organism" often refers to the query, while "target structures" refers to structures from the databases, not the query. Consistency in terminology would improve clarity.

We agree with the reviewer and are now using “query organism” throughout the manuscript instead of “target organism” to be more accurate.

REVIEWER COMMENTS

Reviewer #1 (Remarks to the Author):

The main concern remains - the overall novelty of the pipeline. While the additional analyses have improved the rigor of the workflow, the innovation, novelty, and discovery aspects of the tool are still not evident at a high impact level. Comments on the integration of this on the GEMs and improvement of the GEMs prediction, constraint-based analysis and network-dependent analysis stays without specific response and the new analysis doesn't answer the comment.

We thank the reviewer for their feedback and appreciate the opportunity to further clarify the innovation, novelty, and specific outcomes of our GEM and network-dependent analyses. To clarify the concerns regarding novelty and the specific responses to GEM integration, we would like to highlight the following components already present in the updated manuscript:

1. **Novelty of the Framework**
   While WASP integrates established algorithms (like FoldSeek and SAFE), its primary innovation is the framework itself. High-impact computational biology frequently advances through the novel integration of existing tools to bridge critical gaps, as evidenced by recent highly-cited publications that are essentially tool ensembles lacking de novo wet-lab validation (e.g., https://www.pnas.org/doi/10.1073/pnas.2513219122, https://www.frontiersin.org/journals/bioinformatics/articles/10.3389/fbinf.2025.1684227/full). WASP provides the first highly customizable, single-command solution for whole-proteome structural annotation. We show that it statistically outperforms state-of-the-art sequence tools (like CLEAN and EggNOG) by retrieving "hidden" annotations that sequence homology misses. Furthermore, our application of structure-derived functional profiles to reconstruct phylogenetic trees (Supplementary Note 1) represents a fundamentally novel approach to evolutionary analysis.
2. **GEM Integration and Gap-Filling (Addressed in Revision 1)**
   In response to the previous round of comments, we specifically expanded the GEM analysis to demonstrate how structural homology resolves orphan reactions. Applying WASP to resolve orphan reactions in GEMs introduces a novel approach to metabolic gap-filling. Unlike traditional methods, WASP leverages structural homology to successfully map missing gene-reaction associations. We tested this strategy and validated the improved GEM predictions against *in vivo* essentiality datasets and established literature. The pipeline's ability to identify high-impact targets is evidenced for example by the assignment of Rv2172c to an NADH-dependent MTHFR reaction. This finding aligns with independent biochemical assays (compound AB131), showcasing the tool's discovery potential.
3. **Validation of Discovery Aspects**
   While WASP is computationally driven, its discovery aspect is validated by both complementary functional data and existing literature. For instance, WASP successfully identified novel annotations even within heavily curated model organisms, and these predictions are internally backed up by other functional descriptors, such as CATH structural domain diversity. Furthermore, WASP accurately predicted native candidates for orphan reactions whose mappings are

confirmed by external evolutionary and biochemical literature, such as correctly identifying the bifunctional nature of dethiobiotin synthase in *Y. lipolytica*. In our GEM analysis, this pipeline also identified highly specific, translationally relevant drug targets supported by existing literature (references 50 to 54 in the manuscript - Kelley, E. H. et al. Front. Chem. 12, 1415644 (2024), Sah, S. et al. J. Bacteriol. 202, (2020), Li, J. et al. *ACS Omega* 8, 38406–38417 (2023), Nataraj, V. et al. Mol. Microbiol. 98, 7–16 (2015), López-Agudelo, V. A. et al. PLoS Comput. Biol. 16, e1007533 (2020)).

4. **Impact and Accessibility**
   WASP offers significant time savings and accessibility to non-experts. Without this automated pipeline, researchers would need to manually identify structural homologs, perform individual statistical tests, and manage multiple functional descriptors, such as GO terms, EC numbers, and Pfam domains, separately. This manual process could easily take a week or more for a single proteome, whereas WASP standardizes and automates these steps in a fraction of the time.
   We also addressed previous comments by making the tool easily installable via `pip`, supported by a comprehensive GitHub repository with guidelines, troubleshooting, and example files.

Finally, in our latest release, we have further expanded the discovery potential of WASP by introducing a sequence-agnostic search function. While the initial version of the pipeline focused on integrated structural annotation, this newly implemented feature enables users to explicitly exclude hits that exceed a user-defined sequence-similarity threshold. By masking known sequence homologs, the pipeline is forced to identify remote structural analogs that maintain conserved functional sites despite sharing low sequence identity. This capability targets the "midnight zone" of protein bioinformatics, providing a systematic, automated framework for uncovering novel gene functions that conventional hybrid pipelines or sequence-based tools like EggNOG and BLAST would inherently overlook or filter out.

Reviewer #3 (Remarks to the Author):

The authors have made great efforts to address my previous concerns. I have only one minor point to raise: it would be helpful to define “BH transfer” either in the Figure 3 legend or in the main text (page 9) where it is first introduced.

Otherwise, this is a well-executed study, and the presentation is clear and well written.

We thank the reviewer for the constructive feedback and for the positive assessment of our study. We have addressed this point by adding the abbreviation "BH transfer" on page 9 where it is first introduced in the main text.

Reviewer #3 (Remarks on code availability):

The repository is well documented. By following the instructions, I can install the package easily.
However, when running the example code, I got the following error message:

```
> wasp-run -t 559292

Setting required variables:

Selected taxid is: 559292
Selected evalue threshold is: 1e-10
Selected bitscore threshold is: 50
Traceback (most recent call last):
File ".../wasp-env/bin/wasp-run", line 8, in <module>
sys.exit(main())
^^^^^^
File ".../wasp-env/lib/python3.11/site-packages/wasp/main/run.py", line 73, in main
print(f"Selected max neighbours is: {args.max_n}")
^^^^^^^^^^^
AttributeError: 'Namespace' object has no attribute 'max_n'. Did you mean: 'max_n '?
```

We thank the reviewer for identifying this bug. We have fixed the `AttributeError` in `wasp/main/run.py` by correcting the naming of the `max_n` attribute. Additionally, we have performed a comprehensive review of the codebase to identify and resolve any other potential bugs, ensuring that the example code and package functions run smoothly.

Reviewer #4 (Remarks to the Author):

The authors have substantially improved the manuscript in revision. There are some remaining issues to improve its utility and clarity.

1) The distinction between functional prediction and annotation should be made clear. The name “functional annotation” without the word prediction in the title and tool name is highly misleading. In the examples referred to by the authors, this distinction is made clear. E.g. in https://link.springer.com/article/10.1186/s13059-019-1835-8 explicitly refers to the term “Term-centric prediction of experimental annotations”, and https://www.science.org/doi/10.1126/science.adf2465 uses “Enzyme function prediction” in the title. In these cases, annotation is treated as the benchmarking target or challenge, while the methods themselves generate predictions of annotations.
WASP performs function prediction, or more precisely, prediction of functional annotations, and this should be clearly stated in the tool name, title, and description to accurately convey its scope and to avoid conflating predicted functions with curated or experimentally validated annotations. One cannot simply refer to predictions as annotations, as these are distinct terms with different meanings in the field.

We appreciate the reviewer’s emphasis on the technical distinction between experimental annotation and computational prediction. We recognize that while the term "annotation" is frequently used as a shorthand in high-throughput studies, maintaining a clear separation is vital to avoid misleading users. Consequently, we have updated the manuscript title to “WASP: A pipeline for functional annotation prediction based on AlphaFold structural models” and changed the acronym for WASP to “Whole-proteome Annotation via Structural-

homology Prediction". We have also further revised the text to consistently refer to the outputs of our pipeline as "functional predictions" or "predicted annotations".

2) As this manuscript includes benchmarking analyses, the authors must provide complete and executable scripts to allow accurate reproduction of all analyses and benchmarking comparisons presented in the paper. Although the authors indicate in the code and Software Submission Checklist that these materials are provided, we could not find the corresponding scripts in the GitHub repository or elsewhere. Making these scripts publicly available is essential for reproducibility and for proper evaluation of the study.

We thank the reviewer for their feedback regarding reproducibility and installation. We have now uploaded all executable scripts and respective data files to a dedicated `benchmarks` folder in our GitHub repository. This includes the complete code required to reproduce the benchmarking analyses presented in Figure 3, as well as the specialized scripts and files used to improve and gap-fill the GEMs. We have also performed a comprehensive review of the codebase to identify and resolve additional bugs, ensuring all example scripts run smoothly.

3) A full main figure (Fig. 2) is devoted to a single illustrative example. While this serves as a useful implication of utility, the analysis would be much stronger if the authors generalized this observation across a larger set of protein families. In particular, it would be informative to assess how frequently similar cases occur, and to characterize the features that distinguish such cases from those where simpler approaches suffice
4) More specifically, for the vast majority of cases, sequence homology alone is sufficient for accurate prediction of functional annotations. In such cases, the additional structural prediction step may be unnecessary and computationally wasteful. It would therefore be useful for the authors to provide an analysis offering clearer guidelines on when WASP provides added value over existing homology-based approaches, or to include an evaluation step that determines when sequence homology alone may be insufficient.

We thank the reviewer for these insightful comments. We entirely agree that while sequence homology is often sufficient for closely related proteins, the true value of WASP lies in its ability to rescue annotations in the "twilight zone" of evolutionary divergence where sequence homology fails.

To address both points, we have updated the WASP pipeline to include a new functionality, where users can now filter out sequence homologs above a user-defined sequence identity threshold prior to structural network generation and functional enrichment. We now mention this functionality in the Results (page 5) and in the Methods (page 21).

This allowed us to systematically test WASP's performance when relying *strictly* on distant structural homologs. We re-evaluated the New-392 and Price-149 benchmark datasets under progressively stricter sequence identity exclusion limits (retaining only hits with ≤ 70%, ≤ 50%, and ≤ 30% sequence identity). By filtering out highly identical sequences, we simulated scenarios where traditional sequence-based tools would typically collapse. The results, shown below, demonstrate that WASP is highly robust even when stripped of obvious sequence homologs:

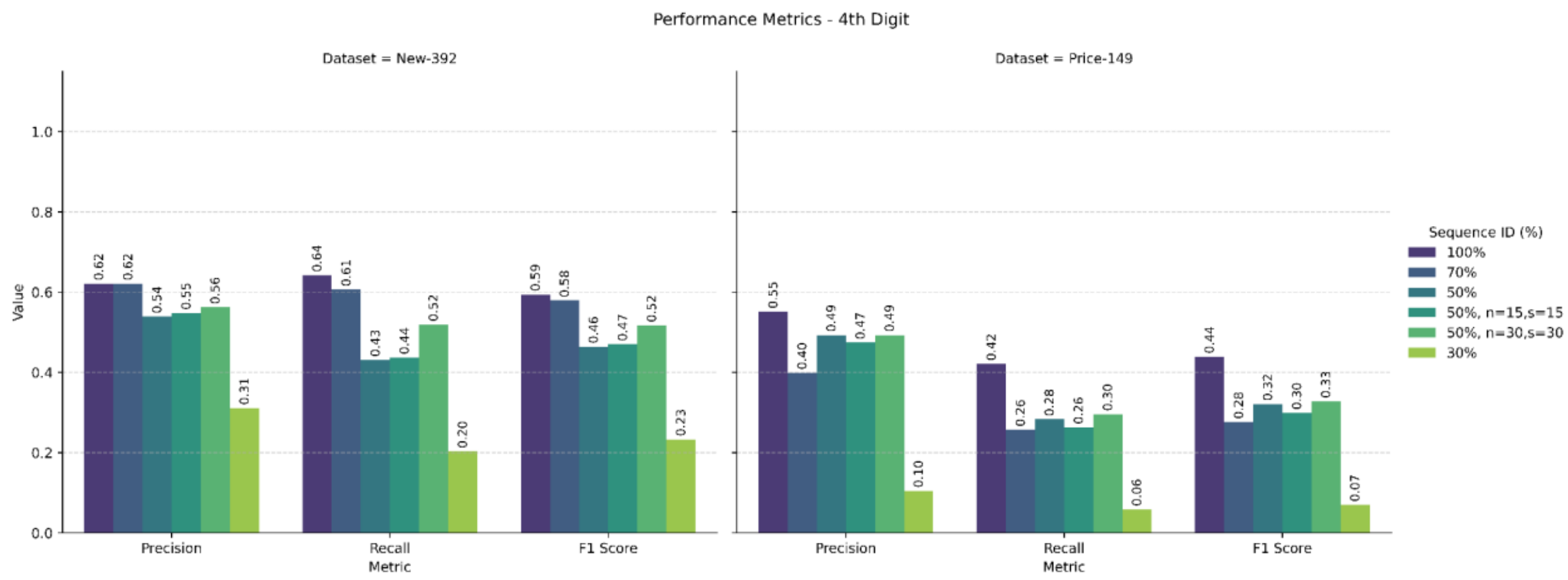

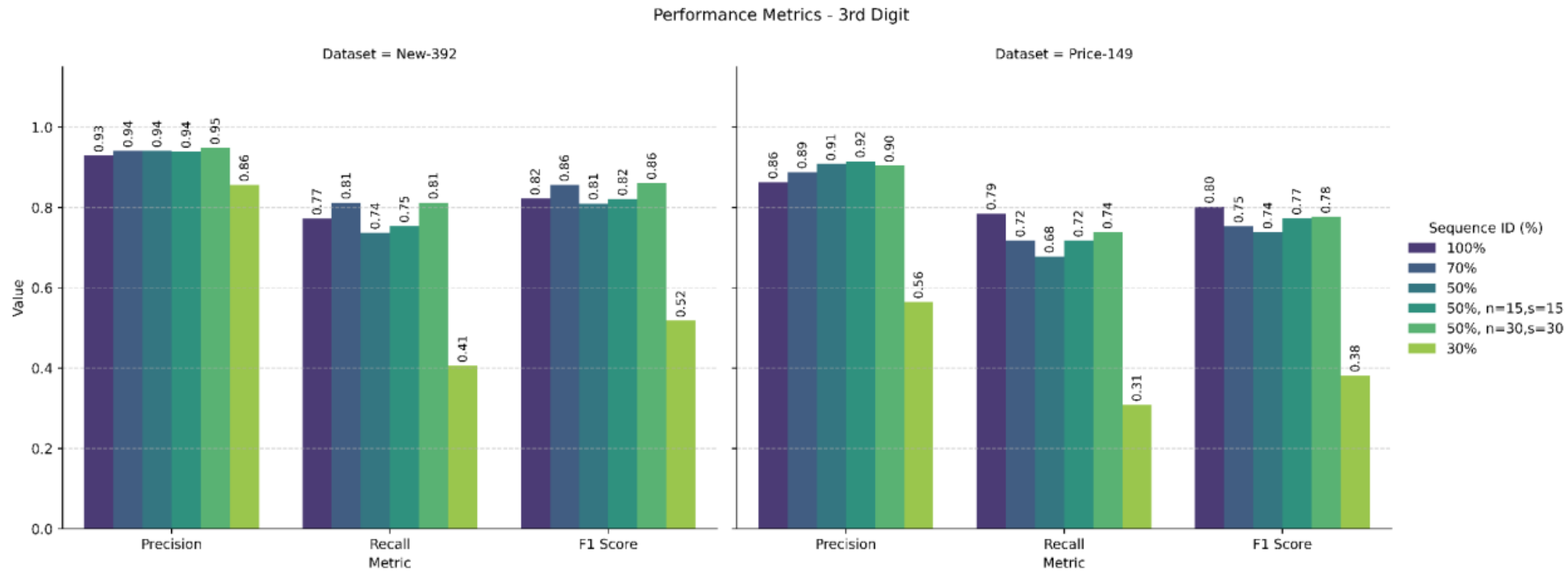

As shown in the figures, excluding hits above 50% or 70% sequence identity does not cause a critical drop in precision and recall in both datasets. A significant drop in performance is only observed when strictly limiting the network to hits with ≤ 30% sequence identity, which aligns with the established biological threshold where structural and functional similarity begins to sharply diverge.

We completely agree with the reviewer that sequence-based homology should remain the primary, computationally inexpensive approach for querying highly conserved proteins (i.e., those with >50% sequence identity). WASP provides its most critical added value specifically for 'orphan' proteins, poorly characterized pathways, and distant evolutionary relationships where sequence identity falls below this 50% threshold. As emphasized in our Discussion regarding the benefits of structural information in annotation pipelines, WASP is designed to complement rather than replace sequence-based tools. Our newly implemented filtering feature is now available for users who wish to bypass redundant sequence matches entirely, allowing them to focus computationally on making novel structural inferences.

Reviewer #4 (Remarks on code availability):

Most critically, the code required to reproduce the analyses presented in the manuscript is not provided, preventing independent validation of the results.
In addition, in this reviewer's opinion, the practical utility of the method is limited by the complexity of installation, which requires numerous non-trivial dependencies and substantial computational resources.

We thank the reviewer for their comments regarding reproducibility and the practical utility of our method. We have addressed these points as follows:

Regarding reproducibility, all executable scripts and data files required to reproduce the benchmarks analyses have been uploaded to the repository. Due to the large file size, the datasets have been hosted as permanent assets in the project's GitHub Release. The data can be accessed here: https://github.com/gioodm/wasp-proteins-annotation/releases/tag/v1.1.1

To address the concerns regarding computational costs and complexity, we want to clarify that the primary requirement for the user is the availability of protein structures and the one-time download and pre-processing of the structural databases. Once this data is in place, the computational cost of running the WASP pipeline is significantly reduced. Furthermore, we emphasize that the primary added value of WASP is the significant time savings for researchers. Without this automated pipeline, a researcher would need to manually identify annotations for similar species, perform individual statistical tests, and manage multiple functional descriptors (e.g., GO terms, EC numbers, and Pfam domains) separately. This manual process could easily take a week or more for a single proteome, whereas WASP standardizes and automates these steps to provide a comprehensive functional map in a fraction of the time.

REVIEWER COMMENTS

Reviewer #4 (Remarks to the Author):

Most concerns have been addressed, and the manuscript is substantially improved. One issue that remains insufficiently resolved is the practical utility of the work. In its current form, the contribution is presented primarily as a tool that integrates existing algorithms, but its functionality depends heavily on the availability and versioning of external methods (e.g., Foldseek). This creates a significant limitation, as the long-term usability of the tool may be compromised by changes in these dependencies, while the conceptual advance beyond integration is relatively modest. To ensure robustness and sustained utility in a rapidly evolving field, the authors should provide a fully reproducible deployment framework, such as a Docker container or a complete, version-pinned Conda environment. This would guarantee consistent installation, reproducibility, and continued usability as underlying tools evolve.

Reviewer #4 (Remarks on code availability):

I was unable to install the tool as provided. The code is not self-contained and instead redirects the user to external dependencies (e.g., Foldseek), without sufficient guidance or integration. This makes installation non-trivial and limits reproducibility.
To address this, the authors should provide a fully self-contained implementation or, at minimum, a clearly documented and version-pinned environment (e.g., Docker or Conda) that includes all required dependencies and ensures straightforward installation and execution.

We thank the reviewer for this suggestion, which can fundamentally improve WASP's accessibility, reproducibility, and long-term usability. We agree that relying on the manual installation of external dependencies like Foldseek and gsutil created an unnecessary bottleneck.
To resolve this, we have implemented a complete, version-pinned Conda environment. The repository now includes an `environment.yml` file that automatically resolves and installs Python 3.10, Foldseek 8, the Google Cloud SDK (`gsutil`), and WASP in a single command.
While the reviewer also suggested Docker, we deliberately chose Conda as our deployment framework to better serve our target user base. WASP is primarily run on High-Performance Computing (HPC) clusters, where bioinformaticians rarely have the root privileges required to deploy Docker containers. Conda natively bypasses this issue while still guaranteeing strict version control. The GitHub repository and `README.md` have been updated to reflect this as the standard installation method.

REVIEWER COMMENTS

Reviewer #4 (Remarks to the Author):

The authors addressed my concerns, except that the current solution still leaves the package fully dependent on the continued availability of the authors' GitHub repository. To ensure long-term accessibility and reproducibility, the software should also be deposited in a versioned, immutable archive (e.g., Zenodo or Figshare) matched to the GitHub release.

Reviewer #4 (Remarks on code availability):

The current solution still leaves the package fully dependent on the continued availability of the authors' GitHub repository. Because GitHub repositories can be altered or removed, I recommend that the editorial team require deposition of a versioned, immutable archive (e.g., Zenodo or Figshare or equivalent) corresponding to the GitHub release to ensure long-term accessibility, reproducibility, and preservation of the software environment.

Thank you for this important point. We have now deposited WASP in Zenodo (https://zenodo.org/records/20617135), and the DOI is linked from our GitHub repository. The software is versioned and permanently archived for long-term accessibility.
